# Criteria for Control and Remission of Respiratory Allergic Disease With Allergen Immunotherapy: A Delphi Consensus

**DOI:** 10.1002/clt2.70191

**Published:** 2026-08-02

**Authors:** Eloína González‐Mancebo, Juan María Beitia Mazuecos, Ana I Tabar Purroy, Mar Gandolfo‐Cano, Adriana Izquierdo‐Domínguez, María José Castillo Marchuet, Cristina Cuevas Bravo, Leticia Domínguez Cereijo, Alicia Enríquez‐Matas, Ignacio Esteban Gorgojo, Adrián Germán Sánchez, Alba Juárez Guerrero

**Affiliations:** ^1^ Unidad de Alergología Hospital Universitario de Fuenlabrada Madrid Spain; ^2^ Immunotherapy Committee of the SEAIC Madrid Spain; ^3^ Servicio de Alergología Hospital Universitario de Navarra Instituto de Investigación en Ciencias de la Salud (IDISNA) Pamplona Spain; ^4^ Servicio de Alergología Hospital Universitario La Paz Madrid Spain; ^5^ Asthma Committee of the SEAIC Madrid Spain; ^6^ Servicio de Alergología Hospital Universitario Consorci Sanitari de Terrasa Grupo de investigación InOIA (investigación en olfato, inmunología y alergia) Unidad Alergo‐Rino Teknon Barcelona Spain; ^7^ Allergic Rhinology and Conjunctivitis Committee of the SEAIC Madrid Spain; ^8^ Consorci Sanitari de Terrasa Terrassa Spain; ^9^ Servicio de Alergología Hospital General Universitario Gregorio Marañón Madrid Spain; ^10^ H. U Virgen Macarena Seville Spain; ^11^ Servicio de Alergología Hospital Universitario 12 de Octubre Madrid Spain; ^12^ Instituto de Investigación sanitaria Hospital 12 de Octubre (imas12) Madrid Spain; ^13^ Hospital General de Villalba Madrid Spain; ^14^ H.G.U de Castellón Castellón de la Plana Spain; ^15^ Consorcio Hospitalario Provincial de Castellón Castellón de la Plana Spain; ^16^ Hospital Comarcal de Vinaroz Vinaroz Spain; ^17^ H.U de Torrejón Torrejón de Ardoz Spain

**Keywords:** allergen immunotherapy, allergic asthma, allergic rhino‐conjunctivitis, control and remission criteria, Delphi method

## Abstract

**Background:**

Allergic rhinitis, conjunctivitis and asthma are prevalent IgE‐mediated respiratory diseases that frequently coexist and impair quality of life. Allergen immunotherapy (AIT) has clinical benefits and potential disease‐modifying effects. Until recently, standardised definitions of disease control and remission were lacking in routine clinical practice, particularly for allergic rhinitis and conjunctivitis. This study aimed to propose preliminary definitions of control and remission in patients receiving AIT and to assess expert consensus using the Delphi methodology.

**Methods:**

A scientific committee developed a survey based on a literature review and expert input, addressing symptoms, medication use, quality of life, exacerbations and tools for objective and subjective assessment. Forty allergists participated in a two‐round Delphi study. Consensus was predefined as ≥ 70% agreement on a 9‐point Likert scale.

**Results:**

Consensus was achieved for all items after two rounds. The panel agreed on proposed definitions of disease control and clinical remission for allergic rhinitis, conjunctivitis and asthma in the context of AIT, integrating symptom severity, medication use, validated questionnaires and objective measures.

**Conclusions:**

This study presents preliminary expert‐consensus definitions of control and remission in allergic rhinitis, conjunctivitis and asthma in patients undergoing AIT. These definitions should not be interpreted as validated clinical criteria or endpoints. As a preliminary proposal, they aim to provide a structured approach for assessing treatment response in clinical practice by integrating symptom burden, medication use, patient‐reported outcomes and objective measures. They should be regarded as an initial conceptual step, and further prospective studies are needed to validate these criteria and confirm their relevance, feasibility and applicability.

## Graphical abstract

Expert consensus using a Delphi methodology proposed definitions of control and remission for allergic rhinitis, conjunctivitis and asthma in the context of allergen immunotherapy. These consensus‐based criteria integrate symptoms, medication use, patient‐reported outcomes and objective measures to support a more structured assessment of treatment response in clinical practice.

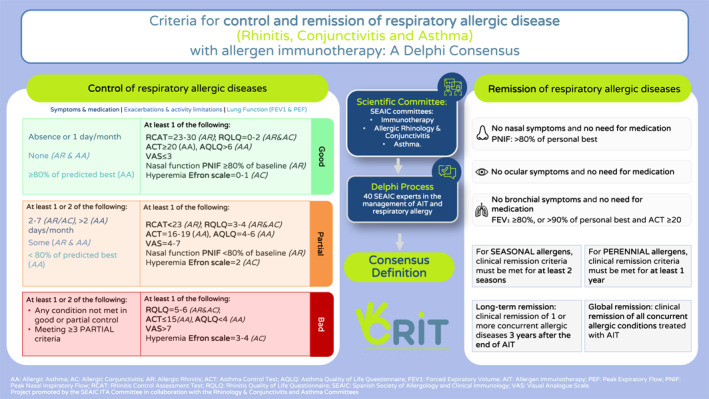

## 1 Introduction

Allergic rhinitis, conjunctivitis and asthma are common chronic inflammatory diseases with prevalence rates of 20%–30% for rhinitis [1], 15%–40% for conjunctivitis [2] and 4%–9% for asthma [1]. These allergic conditions impair quality of life, affecting social, school and work life [3, 4, 5, 6, 7, 8, 9, 10].

These diseases are mediated by allergen‐specific Immunoglobulin E (IgE) and are caused by airborne allergens, which may be seasonal, intermittent or continuous, depending on the nature and intensity of the environmental trigger [4]. The most frequent sensitisers are indoor allergens (mites, fungi and pet dander) and outdoor allergens (pollens and fungal spores) [11].

Allergic rhinitis, conjunctivitis and asthma frequently coexist and should not be viewed as isolated organ‐specific conditions but rather as manifestations of a unified allergic airway disease [12]. This concept of “unified airway disease” highlights the shared pathophysiology and interdependence of the upper and lower airways [3, 4, 13]. This justifies a comprehensive therapeutic approach aimed at controlling symptoms and reducing exposure to causative allergens, primarily through pharmacological treatment and environmental measures, which may be limited or cause adverse effects [14].

However, allergen‐specific immunotherapy (AIT) is assumed to modify key mechanisms of IgE‐mediated respiratory allergy and may help to prevent disease progression. In patients with allergic rhinitis, conjunctivitis and/or asthma, AIT has been associated with improved symptom control, reduced medication uses and better quality of life, with data suggesting long‐term benefits and potential disease‐modifying effects, as well as an acceptable safety and cost‐effectiveness profile [6, 15, 16, 17, 18].

The variability in allergen exposure patterns (perennial and/or seasonal) and the heterogeneity of clinical expression require accurate diagnosis, patient stratification and consideration of patient and caregiver perspectives when AIT is indicated [18, 19]. These factors also affect the assessment of treatment impact. Moreover, the absence of validated efficacy biomarkers contributes to heterogeneity in evaluating treatment outcomes in real‐world practice. Therefore, the implementation of standardised criteria for disease control and remission in clinical settings could support therapeutic decision‐making and facilitate more structured, albeit still exploratory, monitoring of treatment response.

There is currently a paucity of simple, widely accepted criteria to evaluate the effectiveness of AIT in the routine management of respiratory allergy, especially in relation to disease control and remission in allergic rhinitis and conjunctivitis [1, 10, 20, 21, 22, 23, 24, 25, 26, 27, 28, 29]. The EMA guideline on the clinical development of products for specific immunotherapy (CHMP/EWP/18504/2006) is mainly focused on regulatory requirements and proposes efficacy outcome measures for clinical trials, including combined symptom and rescue‐medication scores and various levels of claimed efficacy, from short‐term symptom relief to possible long‐term and disease‐modifying effects [30]. These trial‐orientated outcomes do not necessarily provide directly applicable, operational criteria for control and remission in everyday clinical practice. This lack of alignment between research endpoints and everyday care may be particularly relevant in the era of precision medicine and treat‐to‐target (T2T) strategies.

To investigate this gap in widely accepted criteria, a literature review was conducted. It confirmed the absence of consensus definitions for disease control in allergic rhinitis and conjunctivitis, in contrast with asthma, where standardised control criteria are established in GEMA 5.5 [31] and GINA 2025 [32]. Although ARIA 2016 [33] incorporates the concept of rhinitis control using a global nasal visual analogue scale (VAS) and validated tools such as the Rhinitis Control Assessment Test (RCAT), unified thresholds for remission and their integration with objective measures (e.g., peak nasal inspiratory flow [PNIF] or the Efron grading scale for conjunctival hyperaemia) are still lacking. The remission criteria for allergic asthma are set out in the EUFOREA statement on raising standards in asthma care [34]. During the final stages of manuscript revision, a position paper entitled Clinical Remission in the Practice of Allergy and Clinical Immunology: State of the Art and World Allergy Organization (WAO) Call to Action was published, proposing definitions of remission in allergic rhinitis and asthma. Although this publication became available after the design and completion of the present Delphi process, its overall approach is aligned with the proposals generated in our study [35].

Therefore, the objective of this study was to explore a possible unified definition of control and remission for allergic rhinitis, conjunctivitis and asthma in patients receiving AIT in routine clinical practice using the Delphi process to explore the consensus reached on these criteria by a group of experts. These proposed criteria are intended as an initial exploratory proposal to support clinical evaluation and discussion. Further refinement and validation are required before they can be considered for routine clinical use.

## 2 Methods

### 2.1 Funding and Independence

This project was conceived and organised by members of the Immunotherapy Committee of the Spanish Society of Allergy and Clinical Immunology (SEAIC) and conducted as part of its official scientific activities. Conceptualization and study design, including the development of the questionnaire, were driven exclusively by the members of the Scientific Committee (SC) (most of whom are allergists with more than 20 years of experience in immunotherapy), following approval by the SEAIC Executive Board as an official scientific activity. All authors are members of SEAIC committees (Immunotherapy, Rhinitis and Conjunctivitis and Asthma). First and second author of the manuscript served as study coordinators, overseeing scientific development and co‐ordination, with methodological support for the Delphi process provided by Adelphi Targis, SL.

This project was supported by an unrestricted research grant to SEAIC from the sponsor. The funding covered methodological support provided by Adelphi Targis, SL, as well as administrative and publication‐related costs.

Members of the SC and panellists received remuneration only for the time and work that they devoted to the development of the project, in accordance with standard practice in Delphi studies.

The sponsor had no role in the conceptualization or design of the study, participant selection, questionnaire development, data collection, data analysis, interpretation of results, or preparation of the manuscript.

### 2.2 Study Design

The Delphi method was selected as the most appropriate approach by which to address the study's objective, as it allows the systematic collection and structured synthesis of expert judgments in areas where empirical evidence is limited or heterogeneous or conflicting. In the context of respiratory allergic diseases, particularly regarding criteria for disease control and remission in patients undergoing AIT, the available literature is scarce and lacks standardised definitions. The Delphi is a widely accepted and structured approach technique that therefore provides a rigorous and transparent preliminary proposal by which to identify areas of agreement and disagreement among experts and propose consensus‐based criteria to support clinical assessment.

A modified classic Delphi study was carried out, following the methodological plan described in Table S1. The design, conduct, analysis, and reporting of this Delphi study were guided by the DELPHISTAR recommendations for standardised reporting of Delphi studies (adherence to these guidelines is documented in the completed DELPHISTAR checklist, provided as Table S2).

The study was performed in three phases: (1) From March to September 2024, an online meeting of the SC, composed of 12 members, to discuss the problem, establish the project's scope and draft the Delphi questionnaire. (2) Two successive rounds of anonymous online structured questionnaires with aggregated feedback from panel of experts between rounds to obtain their opinion [36, 37]: (2A) The first round (from 23 October 2024 to 17 November 2024); (2B) The intermediate analysis; (2C) The second round (from 12 December 2024 to 14 January 2025). (3) Analysis and discussion of the results with the SC to reach conclusions (February 2025) (Supporting Information S1: Figure S1).

Consensus on a statement was defined prior to the initiation of both Delphi rounds, as when ≥ 70% of the respondents scored within the three‐point range (1‐3 or 7–9).

### 2.3 Participants

The SC comprised 12 allergologists, most of whom had more than 20 years of experience in immunotherapy from three SEAIC committees: Immunotherapy, Rhinology and Allergic Conjunctivitis and Asthma. The expert panel consisted of 40 specialists with a minimum of 5 years of post‐specialization clinical experience in allergy. The panellists represented clinical practice–based expertise in respiratory allergic diseases and AIT, contributing perspectives derived from routine patient management, long‐term follow‐up, and the practical use of symptom‐based and disease‐control assessment tools in real‐world clinical settings. An initial invitation to participate in the Delphi survey was disseminated through the SEAIC Immunotherapy Committee. Potential experts were informed about the objectives and procedures of the study, drawing on the Committee's membership of allergists actively involved in the clinical management of respiratory allergic diseases and the prescription of AIT. Participation was voluntary. Eligibility was subsequently confirmed through predefined screening criteria embedded in the online questionnaire to ensure appropriate clinical expertise. All experts fulfilling the eligibility criteria who completed the first round were invited to participate in the subsequent round. The screening criteria and eligibility questions are detailed in the questionnaire (Supporting Information S2).

### 2.4 Literature Review and Delphi Questionnaire Development

The questionnaire was developed by the SC based on a structured quasi‐systematic literature review on disease control and remission in allergic respiratory conditions, including rhinitis, conjunctivitis and asthma, with the methodological support of the medical communications consultancy, Adelphi Targis, SL. This additional review was conducted to support the development of the Delphi questionnaire and to identify previously published concepts and criteria related to disease control and remission in allergic rhinitis, conjunctivitis, and asthma in the context of allergen immunotherapy and biologic treatments.

The search was performed in PubMed on 8 March 2024 using predefined search terms related to allergic respiratory diseases, allergen immunotherapy, biologic treatments, and remission. Filters included articles published within the previous 10 years, studies in humans, and publications in English or Spanish.

Original studies, reviews, consensus documents, and clinical guidelines addressing remission or disease control criteria in allergic rhinitis, allergic conjunctivitis, or allergic asthma were considered eligible. Case reports and studies focused on non‐allergic rhinitis, non‐allergic conjunctivitis, or non‐allergic asthma were excluded.

The questionnaire was based on literature criteria (including rating scales) and expert‐driven criteria (Supporting Information S1: Figure S2). During the conceptualization phase, the SC proposed a total of 24 articles. A total of 100 articles were identified through the structured PubMed search strategy. Among these, five articles had already been identified by the experts and were also retrieved through the PubMed search (Total new articles found in PubMed = 100–5 = 95). Study selection was conducted by two reviewers under the supervision of the project coordinators. The detailed search strategy and study selection process are provided in Supporting Information S1: Figure S2.

The questionnaire was divided into 5 blocks: Section 1–Participant profile, Section 2–Allergic rhinitis, Section 3–Allergic conjunctivitis, Section 4–Allergic asthma, Section 5–General concepts (Supporting Information S1: Figure S3). Criteria included in the Delphi questionnaire incorporate daytime/nocturnal symptoms, activity limitation, symptomatic medication use, exacerbations, and both subjective/objective measures: unified VAS (0–10), control questionnaires (RCAT, ACT), quality‐of‐life scores (RQLQ, AQLQ), nasal PNIF, and Efron scale for conjunctival hyperaemia. For asthma, GEMA/GINA control criteria were retained, supplemented with ACT, AQLQ, and FEV1 for consistency.

### 2.5 Delphi Consensus Rounds

This Delphi consensus was conducted in two structured and predefined rounds of questions. The number of Delphi rounds was defined prior to study initiation. In accordance with established Delphi methodology, which commonly involves two to three iterative rounds with analysis following each wave, two rounds were planned. After completion of the second round, a high level of consensus had been achieved across the proposed criteria, and no substantial new areas of disagreement emerged. The pre‐defined number of rounds was therefore considered sufficient to pursue the study objectives, and no additional rounds were deemed necessary [38, 39, 40].

The first Delphi round aimed to collect expert opinions on pre‐defined criteria for control and remission in respiratory allergic diseases, and to assess the initial level of agreement on all proposed items. It comprised 15 descriptive items in Section 1 and 72 items on a Likert‐type scale in Sections 2–5, with some associated open‐ended questions. After completion of the first Delphi round, participants were provided with aggregated feedback summarising the group responses for each item, including the percentages of agreement, disagreement, and neutral responses. Feedback was aggregated across all participants and not differentiated by expert subgroups. The second Delphi round was designed to further explore areas of disagreement by re‐assessing items that did not reach consensus in the first round. The questions on which no consensus (5 items) was reached (< 70% agreement) were repeated in the second round to explore certain disagreements further (Supporting Information S2). Two more items were included in the second round of questions to prioritise definitions with the highest impact on clinical practice. Responses were gathered on a 9‐point Likert scale from 1 (completely disagree) to 9 (completely agree) to define levels of agreement as commonly applied in Delphi studies [38, 39, 41] (Supporting Information S1: Figure S4).

### 2.6 Statistical Analysis

Nominal variables were described by means of frequency. For continuous variables, central tendency and dispersion measurement were calculated. All statistical analyses were performed using IBM SPSS Statistics 27. Charts were subsequently produced using Microsoft Excel. Consensus data were analysed globally. For the interpretation of the Likert‐type scale questions (Sections 2–5), the presentation of the answers was systematised in the range of possible values between 1 and 9 in the following consensus categories: 1–3 = Disagreement, 5–6 = Neutral, and 7–9 = Agreement (Supporting Information S1: Figure S4).

No group‐specific analyses or weighting of expert responses were performed. All participants contributed equally to the analysis, as the panel represented a single professional group with comparable clinical expertise.

## 3 Results

### 3.1 Delphi Panellists' Profile

Initially, 43 experts were invited, 40 of whom completed Round 1 and Round 2 answering all the questions of the survey. The results are therefore from 40 participants, 45% of whom had more than 20 years of experience in managing allergic diseases (Table 1). All panellists were practising physicians who regularly manage patients with allergic diseases, reporting a mean of 175 patients attended per month.

**TABLE 1:** Demographics of the Delphi panellists.

Characteristic	Delphi expert panel (*N* = 40)
	*N* (%)
Gender:	
Male	17 (42.5)
Female	23 (57.5)
Mean age (SD)	51 (± 9.3)
Geographical distribution:	
Andalusia	5 (12.5)
Canary islands	1 (2.5)
Castile‐La Mancha	3 (7.5)
Castile and León	1 (2.5)
Catalonia	4 (10.0)
Community of Madrid	16 (40.0)
Community of Navarre	1 (2.5)
Community of Valencia	3 (7.5)
Extremadura	2 (5.0)
Galicia	1 (2.5)
Balearic islands	1 (2.5)
Basque Country	2 (5.0)
Medical speciality:	
Allergology	40 (100)
Member of the SEAIC immunotherapy committee:	
Yes	31 (77.5)
No	9 (22.5)
Professional experience as responsible for the treatment of allergic disease patients:	
5–10 years	6 (15.0)
11–15 years	7 (17.5)
16–20 years	8 (20.0)
> 20 years	19 (47.5)
Patients with allergic disease seen in the past month *N* (SD)	175 (± 115.2)
Adult patients (%)	68.9
Paediatric patients (%)	31.1
Contribution to clinical studies related to allergic disease or AIT in the past 5 years	
Yes, related to allergic disease	25 (62.5)
Average number (SD)	4.9 (± 4.4)
Yes, related to AIT	32 (80.0)
Average number (SD)	3.6 (± 2.3)
No	3 (7.5)
Contribution as author in publications related to allergic disease or AIT in the past 5 years	
Yes, related to allergic disease	21 (52.5)
Average number (SD)	18.14 (± 24.4)
Yes, related to AIT	22 (55.0)
Average number (SD)	3.64 (± 3.9)
No	10 (25.0)
Teaching activities related to allergic disease or AIT	
Yes	31 (77.5)
No	9 (22.5)
Professional activity:	
Public	23 (57.5)
Private	6 (15.0)
Both	11 (27.5)
Type of health care centre:	
Group 1	5 (12.5%)
Group 2	5 (12.5%)
Group 3	6 (15.0%)
Group 4	7 (17.5%)
Group 5	17 (42.5%)
Immunotherapy unit in the centre^a^:	
Yes	30 (75.0%)
No	10 (25.0%)

*Note:* Group 1: District hospital with fewer than 150 beds on average and low complexity. Group 2: Basic general hospital, medium‐sized with fewer than 200 beds, somewhat higher complexity than Group 1. Group 3: Area hospital, medium‐sized, with about 500 beds, and medium complexity. Group 4: Large hospital, heterogeneous in resources, size and activity, with high teaching intensity and high complexity. Group 5: Hospital with a large structure and high activity, with a comprehensive range of services; including large hospital complexes. https://www.sanidad.gob.es/estadEstudios/estadisticas/docs/CMBD/CLASIFICACIONHOSPITALESCLUSTER.pdf.

Abbreviations: AIT: allergen‐specific immunotherapy; SD: standard deviation; SEAIC (*Sociedad Española de Alergología e Inmunología Clínica*) Spanish Society of Allergology and Clinical Immunology.

^a^ https://www.seaic.org/profesionales/acreditacion‐de‐unidades/acreditacion‐de‐unidades‐de‐inmunoterapia (ACREDITACIONES 2023).

### 3.2 Two Delphi Survey Rounds

In the first round, agreement (score 7–9 on the 9‐point Likert scale) was achieved on 67 items (93%) out of the 72 items in Sections 2–5. The remaining five items, without agreement, were re‐evaluated in the second round together with two new items to investigate a further two questions in greater depth. Consensus was ultimately reached in all items after the two rounds (Table 2). Therefore, a final consensus on all 72 items was reached after the two rounds.

**TABLE 2:** Results of the two rounds of the Delphi process in statements about the criteria for defining control and remission of allergic rhinitis, conjunctivitis and asthma.

Statement/Item	Round 1 agreement *N* (%)	Round 2 agreement *N* (%)
SECTION 2: Allergic rhinitis		
Good control of allergic rhinitis		
RCAT score (6–30) between 23 and 30	33 (82.5)	—
Absence of daytime symptoms of allergic rhinitis (nasal itching, nasal congestion, rhinorrhoea, sneezing)	36 (90.0)	—
Presence of daytime symptoms of allergic rhinitis on less than 2 days per month (nasal itching, nasal congestion, rhinorrhoea, sneezing)	35 (87.5)	—
Absence of nighttime symptoms of allergic rhinitis (nasal itching, nasal congestion, rhinorrhoea, sneezing)	37 (92.5)	—
Nasal symptoms measured by VAS (0–10) ≤ 3 cm (nasal itching, nasal congestion, rhinorrhoea, sneezing)	34 (85.0)	—
RQLQ score (0–6) of 0–2	35 (87.5)	—
No limitations on the patient's daily activities (e.g., routine tasks at work, school or home)	37 (92.5)	—
No use of symptomatic medication	33 (82.5)	—
Use of symptomatic medication on less than 2 days per month	34 (85.0)	—
Nasal function/patency indicated by PNIF > 80% compared to baseline	29 (72.5)	—
Absence of exacerbations	34 (85.0)	—
*Considering exacerbation as the reappearance of symptoms that affect quality of life and require symptomatic treatment following exposure to the allergen for which the patient is undergoing allergen immunotherapy (AIT).*		
Meeting ALL the criteria described above for good control is necessary to define allergic rhinitis as well‐controlled	33 (82.5)	—
After reaching a consensus on the criteria for good control of allergic rhinitis in Round 1, the SC members developed the following definition, which was submitted to consensus in Round 2 to establish a definition applicable to clinical practice.		28 (70.0)
To claim *good control of allergic rhinitis*, all the clinical criteria must be met:		
*Absence of daytime symptoms* of allergic rhinitis (nasal itching, nasal congestion, rhinorrhoea, sneezing) or presence of these symptoms on *less than 2 days per month*.		
*Absence of nighttime symptoms* of allergic rhinitis (nasal itching, nasal congestion, rhinorrhoea, sneezing).		
*No limitations on the patient's daily activities* (e.g., routine tasks at work, school or home).		
*Absence of symptomatic medication* or use on *less than 2 days per month*.		
*Absence of exacerbations*.		
And at least one of the following:		
*RCAT (6‐30) between 23 and 30*.		
Nasal symptoms measured by *VAS (0‐10) ≤ 3 cm* (nasal itching, nasal congestion, rhinorrhoea, sneezing).		
*RQLQ* quality of life questionnaire *score (0‐6)* of *0‐2*.		
Nasal function/patency indicated by a *PNIF > 80%* relative to baseline.		
Partial control of allergic rhinitis		
RCAT (6–30) score < 23	27(67.5)*	28 (70.0)
Presence of daytime symptoms of allergic rhinitis on 2–7 days per month (nasal itching, nasal congestion, rhinorrhoea, sneezing)	36 (90.0)	—
Any nighttime symptoms of allergic rhinitis (nasal itching, nasal congestion, rhinorrhoea, sneezing)	35 (87.5)	—
Nasal symptoms measured by a 4–7 cm VAS (0–10) (nasal itching, nasal congestion, rhinorrhoea, sneezing)	33 (82.5)	—
RQLQ (0–6) score of 3–4	35 (87.5)	—
Some limitations on the patient's daily activities (e.g., routine tasks at work, school or home)	31 (77,5)	—
Use of symptomatic medication on 2–7 days per month	34 (85.0)	—
Nasal function/patency indicated by a PNIF < 80% of baseline	30 (75.0)	—
Having one or more exacerbations per year	30 (75.0)	—
*Considering exacerbation as the reappearance of symptoms that affect quality of life and require symptomatic treatment following exposure to the allergen for which the patient is undergoing allergen immunotherapy (AIT).*		
Meeting LESS THAN 3 of the previously described criteria for partial control is necessary to define allergic rhinitis as partially controlled	28 (70.0)	—
Poor control of rhinitis		
Any condition not met within the criteria for good or partial control should be considered as indicating poorly controlled allergic rhinitis	30 (75.0)	—
The presence of 3 or more characteristics of partially controlled rhinitis is necessary to define allergic rhinitis as poorly controlled	28 (70.0)	—
Nasal symptoms measured by VAS (0–10) ≥ 7 cm (nasal itching, nasal congestion, rhinorrhoea, sneezing)	37 (92.5)	—
RQLQ score (0–6) of 5–6	38 (95.0)	—
Clinical remission of rhinitis		
Absence of nasal symptoms (nasal itching, nasal congestion, runny nose, sneezing)	39 (97.5)	—
No need for symptomatic treatment	37 (92.5)	—
Nasal function indicated by PNIF > 80% of personal best.	35 (87.5)	—
SECTION 3: Allergic conjunctivitis		
Good control of allergic conjunctivitis		
Absence of ocular symptoms (itching, excessive tearing, conjunctival erythema)	37 (92.5)	—
Presence of ocular symptoms on less than 2 days per month (itching, excessive tearing, visual discomfort) *	37 (92.5)	—
Ocular symptoms measured by VAS (0–10) ≤ 3 cm*	37 (92.5)	—
RQLQ score (0–6) of 0–2	38 (95.0)	—
Level of hyperaemia assessed on the Efron scale^a^ (0–4) of 0–1.	35 (87.5)	—
Absence of symptomatic medication use	31 (77.5)	—
Use of symptomatic medication on less than 2 days per month	35 (87.5)	—
Meeting ALL the previously described criterion for good control is necessary to define allergic conjunctivitis as well‐controlled	33 (82.5)	—
After reaching a consensus on the criteria for good control of allergic conjunctivitis in Round 1, the SC members produced the following definition, which was submitted to consensus in Round 2 to establish a definition applicable to clinical practice.		33 (82.5)
To claim *good control of allergic conjunctivitis*, all the clinical criteria must be met:		
*Absence of ocular symptoms* (itching, watering, visual discomfort) or presence of these symptoms on less *than 2 days per month*.		
*Absence* of *symptomatic medication* use or use them on *less than 2 days per month*.		
And at least one of the following:		
Ocular symptoms measured by *VAS* (0–10) *≤ 3 cm*.		
*RQLQ* score (0–6) of *0‐2*.		
Level of hyperaemia assessed on the *Efron scale* (0–4) of *0‐1*.		
Partial control of allergic conjunctivitis		
Presence of ocular symptoms on between 2 and 7 days per month (itching, excessive tearing, visual discomfort)	37 (92.5)	—
Ocular symptoms measured by a 4–7 cm VAS (0–10)	35 (87.5)	—
RQLQ score (0–6) of 3–4	36 (90.0)	—
Level of hyperaemia assessed on the Efron scale (0–4) of 2	31 (77.5)	—
Use of symptomatic medication on between 2 and 7 days per month	36 (90.0)	—
Meeting LESS THAN 3 of the previously described criteria for partial control is necessary to define allergic conjunctivitis as partially controlled	30 (75.0)	—
Poor control of allergic conjunctivitis		
Any condition not met within the criteria for good or partial control should be considered as poorly controlled allergic conjunctivitis	32 (80.0)	—
The presence of three or more characteristics of partially controlled conjunctivitis is necessary to define allergic rhinitis as poorly controlled	32 (80.0)	—
Ocular symptoms measured by VAS (0–10) > 7 cm	38 (95.0)	—
RQLQ score (0–6) of 5–6	38 (95.0)	—
Level of hyperaemia assessed on the Efron scale (0–4) of 3–4	35 (87.5)	—
Clinical remission of allergic conjunctivitis		
Absence of ocular symptoms and signs	40 (100)	—
No need for symptomatic treatment	37 (92.5)	—
SECTION 4: Allergic asthma		
Good control of allergic asthma		
All the following criteria must be met:	38 (95.0)	—
No daytime symptoms or ≤ 2 days per month		
No activity limitations		
No nighttime symptoms		
Use of rescue medication: None or ≤ 2 days per month		
Lung function:		
FEV_1_ ≥ 80% of predicted best		
PEF ≥ 80% of personal best		
No exacerbations.		
Allergic asthma symptoms measured with a score ≤ 3 cm on the VAS scale (0–10)	37 (92.5)	—
ACT (5–25) score ≥ 20	28 (95.0)	—
AQLQ (1–7) score > 6	36 (90.0)	—
Partial control of allergic asthma		
Any of the following criteria must be met:	35 (87.5)	—
Daytime symptoms > 2 days per month		
Any activity limitations		
Any nighttime symptoms		
Use of rescue medication > 2 days per month		
Lung function:		
FEV_1_ < 80% of predicted best		
PEF < 80% of personal best		
Exacerbations: 1 or more per year		
Allergic asthma symptoms measured with a score of 4–7 cm on the VAS scale (0–10)	35 (87.5)	—
ACT Score (5–25) of 16–19	38 (95.0)	—
AQLQ score (1–7) of 4–6	36 (90.0)	—
Poor control of allergic asthma		
Three or more characteristics of partially controlled asthma are present	37 (92.5)	—
Allergic asthma symptoms with a score > 7 cm on the VAS scale (0–10)	37 (92.5)	—
ACT Score (5–25) ≤ 15	39 (97.5)	—
AQLQ score (1–7) < 4	36 (90.0)	—
Clinical remission of allergic asthma		
All the following criteria must be met:	37 (92.5)	—
Controlled asthma (ACT ≥ 20)		
No need for relief or rescue medication		
No exacerbations and no need for systemic steroid courses or maintenance treatment		
Spirometry with FEV_1_ ≥ 80%, or, in previous studies, values > 90% of personal best		
Spirometry with negative bronchodilator test.		
SECTION 5: General concepts		
Patient satisfaction with AIT treatment		
Usefulness of using the ESPIA (*Satisfaction Scale for Patients Receiving Allergen Immunotherapy*) questionnaire^b^ to assess patient satisfaction with AIT in clinical practice	33 (82.5)	—
Required duration of clinical remission criteria for allergic diseases (allergic rhinitis, allergic conjunctivitis and/or allergic asthma)		
The minimum periods for assessing clinical remission are different for perennial and seasonal allergens	31 (77.5)	—
For seasonal allergens, the clinical remission criteria must be met for at least one season	24 (60.0) *	—
For seasonal allergens, the clinical remission criteria must be met for at least two seasons	—	**39 (97.5)**
For perennial allergens, the clinical remission criteria must be met for at least 6 months	21 (52.5) *	—
For PERENNIAL allergens, the clinical remission criteria must be met for at least 1 year	—	**35 (87.5)**
Global remission of allergic diseases (allergic rhinitis, allergic conjunctivitis and/or allergic asthma)		
Global remission is defined as the clinical remission of all concurrent allergic conditions treated with AIT	38 (95.0)	—
The minimum periods for assessing global remission are different for perennial and seasonal allergens	33 (82.5)	—
For seasonal allergens, the GLOBAL remission criteria must be met for at least one season	22 (55.0) *	—
For SEASONAL allergens, the GLOBAL remission criteria must be met for at least two seasons	—	**38 (95.0)**
For perennial allergens, the GLOBAL remission criteria must be met for at least 6 months	22 (55.0) *	—
For PERENNIAL allergens, the GLOBAL remission criteria must be met for at least 1 year	—	**34 (85.0)**
Long‐term remission of allergic diseases (allergic rhinitis, allergic conjunctivitis and/or allergic asthma)		
Long‐term remission is defined as the maintenance of clinical remission of one or more concurrent allergic diseases 1 year after the end of AIT treatment	13 (32.5) *	—
Long‐term remission is defined as the maintenance of clinical remission of one or more concurrent allergic diseases 3 years after the end of AIT treatment	33 (82.5)	

*Note:* In bold and with asterisk (*): no consensus was reached in Round 1. In grey shading: new items asked about in Round 2.

Abbreviations: ACT: Asthma Control Test; AIT: allergen immunotherapy; AQLQ: Asthma Quality of Life Questionnaire; FEV_1_: Forced Expiratory Volume; PEF: Peak Expiratory Flow; PNIF: Peak Nasal Inspiratory Flow; RCAT: Rhinitis Control Assessment Test; RQLQ: Rhinoconjunctivitis Quality of Life Questionnaire; SC: Scientific Committee; VAS: Visual Analogue Scale.

^a^ Sánchez‐Hernández M, et al. Consensus Document on Allergic Conjunctivitis (DECA). J Investig Allergol Clin Immunol 2015; 94–106.

^b^ Justicia JL, et al. Validation of the first treatment‐specific questionnaire for the assessment of allergic patients' satisfaction with their allergen‐specific immunotherapy: the ESPIA questionnaire. J Allergy Clin Immunol; 131. Epub ahead of print 2013. DOI: 10.1016/J.JACI.2012.11.049.

### 3.3 Consensus on Definitions of Allergic Diseases

Table 2 displays each questionnaire item together with the agreement percentages achieved in Rounds 1 and 2 of the Delphi process. It is divided into separate sections showing the items for control (good, partial, and poor control) and clinical remission of allergic rhinitis, conjunctivitis and asthma.

- Symptomatic treatment includes any rescue or maintenance medication used by the patient; AIT is not regarded as symptomatic treatment.
- Clinical remission is present when the patient is asymptomatic and does not require symptomatic medication, and it may occur while the patient is receiving AIT or after its completion.
- The minimum duration of clinical remission varies according to the type of allergen:
  ◦ Perennial: more than 1 year without symptoms or symptomatic medication.
  ◦ Seasonal: at least 2 consecutive seasons without symptoms or symptomatic medication.
- Global allergic disease remission is achieved when all three allergic diseases considered are simultaneously in clinical remission.
- Long‐term remission requires that this remission status be maintained for at least 3 years after AIT completion.
- Clinical remission during AIT treatment represents a distinct, earlier phase than long‐term remission, which requires maintenance of remission for 3 years after AIT completion.

After Round 1, the SC integrated the item‐level consensus results into composite definitions of control and remission for each condition. These composite definitions were subsequently submitted to the expert panel in Round 2, where consensus was achieved. These definitions of good, partial and poor control, as well as definitions of clinical remission for the three allergic conditions established by consensus are shown in Tables 3, 4, 5.

**TABLE 3:** Criteria for allergic rhinitis control and remission achieved with AIT.

Item	Control of allergic rhinitis			Remission of allergic rhinitis
	Good control	Partial control	Poor control	
	All clinical criteria	1 or 2 of the following criteria	Any of the following criteria	
Daytime rhinitis symptoms	Absence or 1 day per month	Between 2 and 7 days per month	Any condition not met within the criteria for good or partial control should be considered as indicating poor control of allergic rhinitis. Meeting three or more criteria of partially controlled rhinitis.	Absence of nasal symptoms
Nighttime rhinitis symptoms	None	Some		
Activity limitations	None	Some		
Use of symptomatic medication	Absence or 1 day per month	Between 2 and 7 days per month		Absence of need for symptomatic treatment
Exacerbations	None	1 or more per year		
+ Meeting at least one of the following questionnaires/scales:				
RCAT score (6–30)	Between 23 and 30	< 23		
Nasal symptoms measured by VAS (0–10 cm)	≤ 3	From 4 to 7	> 7	
RQLQ score (0–6)	From 0 to 2	From 3 to 4	From 5 to 6	
Nasal function/patency as indicated by PNIF**^a^** score	≥ 80% of baseline	< 80% of baseline		> 80% of personal best

Abbreviations: RCAT: Rhinitis Control Assessment Test; RQLQ: Rhinitis Quality of Life Questionnaire; VAS: Visual Analogue Scale.

^a^ PNIF: Peak Nasal Inspiratory Flow (Based on Valero A. et al. Position paper on nasal obstruction: evaluation and treatment. J Investig Allergol Clin Immunol 2018; 28: 67–90).

**TABLE 4:** Criteria for allergic conjunctivitis control and remission achieved with AIT.

Item	Control of allergic conjunctivitis			Remission of allergic conjunctivitis
	Good control	Partial control	Poor control	
	All clinical criteria	1 or 2 of the following criteria	Any of the following criteria	
Ocular symptoms	Absence or 1 day per month	Between 2 and 7 days per month	Any condition not met within the criteria for good or partial control should be considered as indicating poor control of allergic conjunctivitis. Meeting three or more criteria of partially controlled conjunctivitis.	Absence of ocular symptoms and signs.
Use of symptomatic medication	Absence or 1 day per month	Between 2 and 7 days per month		No need for symptomatic treatment
+ Meeting at least one of the following questionnaires/scales:				
Ocular symptoms measured by VAS (0–10 cm)	≤ 3	Between 4 and 7	> 7	
RQLQ score (0–6)	From 0 to 2	From 3 to 4	From 5 to 6	
Degree of hyperaemia assessed using the Efron scale (0–4)^a^	Grade 0 to 1	2	Grade 3 to 4	

Abbreviations: RQLQ: Rhinitis Quality of Life Questionnaire; VAS: Visual Analogue Scale.

^a^ Efron scale: based on Sánchez‐Hernández M, et al. Consensus Document on Allergic Conjunctivitis (DECA). J Investig Allergol Clin Immunol 2015; 94–106.

**TABLE 5:** Criteria for allergic asthma control and remission achieved with AIT.

Item^a^	Control of allergic asthma			Remission of allergic asthma
	Good control	Partial control	Poor control	
	All the following:	Any of the following:	3 or more of the following:	Definition of clinical remission^b^
Daytime symptoms	None or ≤ 2 days per month	> 2 days per month	> 2 days per month	No need for relief or rescue medication. No exacerbations and no need for cycles of systemic steroid or inhaled maintenance treatment. Spirometry with FEV1 ≥ 80%, or in previous years > 90% of personal best. Controlled asthma (ACT ≥ 20).
Nighttime symptoms	None	Any	Any	
Activity limitations	None	Any	Any	
Use of rescue medication	None or ≤ 2 days per month	> 2 days per month	> 2 days per month	
Lung function:				
FEV_1_	≥ 80% of predicted best	< 80% of predicted best	< 80% of predicted best	
PEF	≥ 80% of personal best	< 80% of personal best	< 80% of personal best	
Exacerbations	None	≥ 1/year	≥ 1/year	
+ Meeting at least one of the questionnaires/scales				
ACT Score (5–25)	≥ 20	From 16 to 19	≤ 15	
Symptoms of allergic asthma measured using the VAS scale (0–10 cm)	≤ 3	From 4 to 7	> 7	
AQLQ score (1–7)	> 6	From 4 to 6	< 4	

Abbreviations: ACT: Asthma Control Test; AQLQ: Asthma Quality of Life Questionnaire; FEV_1_: Forced Expiratory Volume; PEF: Peak Expiratory Flow; VAS: Visual Analogue Scale.

^a^ Items based on GEMA criteria: *Guía española para el manejo del asma GEMA5.3*, https://www.semg.es/images/2023/documentos/GEMA_53.pdf.

^b^ Items based on ref. [29].

Overall, the highest levels of agreement were observed for symptom‐based definitions and criteria for the use of medication in the three diseases. Items related to the minimum duration required further clarification during Round 2 before consensus was achieved. The high overall agreement reflects conceptual alignment among experts, although these definitions remain exploratory and require prospective validation.

## 4 Discussion

This study explores the lack of agreed, operational definitions for disease control and remission in allergic rhinitis, conjunctivitis and asthma in the specific context of AIT in the clinical setting. Using a structured Delphi process, expert consensus was reached on a set of proposed definitions that may help to support a more uniform assessment of response to treatment. Rather than introducing new outcome measures, this work brings together and refines existing concepts into a preliminary approach that could be considered for routine AIT follow‐up, pending further validation in future studies.

Unlike other complex medical conditions, such as chronic urticaria and rheumatological diseases, allergic rhinitis and conjunctivitis have lacked clear definitions of control and/or remission, despite their high prevalence. This gap is particularly relevant in view of the clinical heterogeneity of these conditions, which is influenced by allergen type, seasonal or perennial exposure patterns, and frequent multimorbidity [42]. Such variability may complicate the interpretation of treatment response and indicates a possible need for structured assessment criteria.

The T2T concept guides the management of chronic inflammatory disease by defining clear, measurable therapeutic goals (such as remission or low disease activity) with regular monitoring and treatment adjustments. Originating in endocrinology, it expanded to rheumatology and type 2 inflammation (asthma, atopic dermatitis), reducing structural damage, morbidity, and costs by means of tight control and shared decisions. It could be hypothesised that the T2T strategy is applicable to the evaluation of the clinical benefits of AIT in patients with respiratory allergy (allergic rhinitis, conjunctivitis and/or asthma). Such an approach may be of interest, as AIT not only can improve symptoms but also has potential disease‐modifying effects, with treatment outcomes being measured by defined clinical objectives.

The assessment of the efficacy of AIT shows notable differences between clinical trials and daily clinical practice in several key areas. Clinical trials rely on standardised, pre‐defined efficacy measures, such as combined symptom and rescue medication scores, to rigorously evaluate specific endpoints, including short‐term relief and long‐term disease‐modifying effects [30], whereas daily practice often uses more subjective tools, such as patient‐reported outcomes, symptom diaries, and overall satisfaction, which may lead to greater variability in the perception of success. The differences in assessment methods between clinical trials and everyday clinical settings highlight the likely usefulness of standardised, practical criteria that could help bridge the gap between research results and the real‐world assessment of the benefit of AIT. Once the patient is receiving effective, evidence‐based treatment, it could be very useful to have accessible tools that help to assess the effectiveness of treatment more easily in clinical practice.

The Delphi process achieved agreement on all aspects evaluated, reflecting a high level of agreement among the participating experts. To support the reliability of the findings, responses were collected and analysed anonymously, and free‐text comments were independently reviewed by two researchers. The central findings can be summarised as follows: Definitions of good, partial, and poor disease control have been proposed for allergic rhinitis, conjunctivitis and asthma, together with criteria for global and long‐term remission. It should be noted that this is the first consensus to explicitly propose remission criteria for allergic rhinitis and conjunctivitis, while maintaining the established criteria for asthma. The proposed control criteria suggest incorporating not only symptoms and medication use, but also symptom scale results, quality‐of‐life scores, and functional measures. In addition, the consensus supports the value of the ESPIA questionnaire for assessing patients' satisfaction with AIT.

The proposed remission criteria (absence of nasal symptoms, no need for symptomatic treatment, and > 80% of personal best nasal function/patency (as indicated by PNIF score) align broadly with prior expert statements, including those by Lommatzsch [43], which require minimal or no symptoms (VAS < 2), no exacerbations, and no systemic steroids for allergic rhinitis treatment (all fulfiled for at least 1 year). Regarding allergic asthma remission criteria, our consensus aligns with the “EUFOREA statement about raising the bar in asthma care” [34], adapted to the specific context of AIT. Interestingly, a recently published international position paper on clinical remission in allergy and clinical practice proposed conceptual approaches for remission in allergic rhinitis and asthma that are broadly consistent with the definitions generated in the present Delphi study. This convergence may support the clinical relevance of the domains identified by our expert panel, including symptom control, medication use, patient‐reported outcomes, and objective assessment measures [35].

However, the present definitions should be interpreted as expert‐endorsed proposals rather than validated clinical endpoints, as their predictive value for long‐term outcomes has not yet been prospectively assessed.

Regarding their application in clinical practice, these definitions could be applied at specific points during AIT, including the initial assessment before AIT, during follow‐up visits (every 6–12 months) and end‐of‐treatment assessment. It is expected that their use could structure documentation of the response to treatment and facilitate shared decision‐making, especially when considering the continuation, modification, or discontinuation of treatment. However, their routine application may pose practical difficulties. The systematic use of multiple questionnaires, repeated functional measurements, and quality‐of‐life instruments requires additional time, organisational resources, and, in some settings, financial investment. In high‐volume outpatient clinics, completing and scoring several PROs at each visit may be difficult, and access to objective tests may be limited by availability, costs, or the need for specialised staff and equipment. These feasibility constraints may limit implementation in routine care and should be considered when translating the consensus into daily practice. Nevertheless, it is preferable to have defined criteria that can be used, whenever possible, rather than to lack them. It is important to note that it remains unclear whether the systematic use of these definitions would directly lead to the optimization of treatment strategies or improved patient outcomes. These benefits are currently hypothetical and require confirmation through prospective real‐world studies of different healthcare systems, evaluating clinical impact, feasibility, and cost‐effectiveness.

As with all Delphi surveys, the findings reflect expert consensus, which defines their limitations and underscores the need for subsequent confirmation. The Delphi panel was intentionally composed of experienced allergists, as AIT is a specialised treatment administered by physicians with specific expertise in allergic diseases; consequently, the consensus reflects the perspective of clinicians with established expertise in allergies and AIT. However, because experts were selected through a national scientific society committee, the panel may have reflected a relatively homogeneous perspective. Patient representatives were not included in the Delphi panel, as the aim was to establish clinical guidance definitions based on expert knowledge and current clinical practice from the perspective of allergists with expertise in respiratory allergy and AIT. The study was conducted within the Spanish healthcare setting, where allergists are the specialists primarily responsible for the indication, prescription, and follow‐up of AIT. We fully recognize that the organization of allergy care differs across European healthcare systems and that, in some countries, AIT is managed by other specialists with specific training and expertise in allergy. Another limitation is that the study was conducted in a single country, which may limit generalizability and therefore makes external validation in different health systems necessary. Nevertheless, the high level of agreement achieved suggests consistency in expert opinion. Future international and multidisciplinary initiatives, ideally including patient participation, will be valuable to further refine and validate these proposed definitions.

In conclusion, this Delphi‐based expert consensus proposes a preliminary set of structured, clinically orientated criteria for defining disease control and remission in allergic rhinitis, conjunctivitis and asthma in the context of AIT treatment. By integrating symptom burden, medication use, patient‐reported outcomes, and selected objective measures, these criteria may contribute to a more consistent assessment of treatment response in routine practice and support a more structured follow‐up and shared decision‐making.

These consensus‐based criteria may also support a more structured assessment of treatment response, complementing clinical judgement and existing guideline recommendations. However, they should not be interpreted as validated clinical endpoints, since their predictive value, reliability, and impact on long‐term outcomes have not yet been prospectively established. In addition, implementation in routine care may be challenged by feasibility barriers, including questionnaire burden, repeated functional assessments, resource availability, and access to objective testing in high‐volume or resource‐limited settings. Prospective validation across different healthcare settings will be essential to determine its reliability and real‐world applicability. Until such evidence is available, these criteria should be regarded as a preliminary proposal intended to stimulate scientific dialog and to contribute to ongoing efforts to refine the evaluation of patients undergoing AIT.

## Author Contributions

E.G.M. and J.M.B.M. developed the conceptualisation and design of the study, carried out the analysis and interpretation of data, and were responsible for the development of the manuscript. A.I.T.P., M.G.C. and A.I.D. made substantial contributions to the conception and design, analysis and interpretation of data, and contributed to the development of the manuscript. M.J.C.M., C.C.B., L.D.C., A.E.M., I.E.G., A.G.S. and A.J.G. reviewed the study design and the interpretation of data and critically revised the manuscript. All authors have given final approval of the version to be published. They agree to be accountable for all aspects of the work in ensuring that questions related to the accuracy or integrity of any part of the work are appropriately investigated and resolved.

## Funding

This project was supported by an unrestricted research grant from Allergy Therapeutics Ibérica, S.L.U. to the Spanish Society of Allergy and Clinical Immunology (SEAIC). The funding covered methodological support provided by Adelphi Targis, SL, as well as administrative and publication‐related costs. Members of the Scientific Committee and panellists received compensation strictly limited to remuneration for the time and work devoted to the development of the project, in accordance with standard practice in Delphi studies. Allergy Therapeutics Ibérica, SL had no role in the conceptualisation or design of the study, participant selection, questionnaire development, data collection, data analysis, interpretation of results, or preparation of the manuscript. All scientific decisions were made independently by the authors.

## Ethics Statement

This study consisted of a structured Delphi survey conducted exclusively among healthcare professionals recognised as experts in the field, who were asked to provide their opinions on specific issues related to allergy and immunotherapy. No patients were involved, and no patient‐level data, biological samples, or identifiable personal data were collected. The need for formal ethics committee approval was assessed in accordance with applicable local regulations governing research involving human participants. As the study involved a voluntary, anonymous survey of healthcare professionals and did not include clinical interventions or the collection of sensitive personal data, formal ethics committee review was not required. In accordance with the ISPOR Good Practices for Outcomes Research (2017), specifically Sections 9.2 and 9.3 from Appendix no. 9, and the EPHMRA Code of Conduct (2023), specifically Sections 1.2 and 1.3, as well as examples from similar published studies, ethical review is not required for this type of non‐interventional study. All participants were informed about the purpose and procedures of the study prior to participation and provided electronic informed consent through the online survey platform. Responses were collected and analysed anonymously, and confidentiality was maintained throughout the study. The study was conducted in accordance with applicable professional and research conduct standards.

## Conflicts of Interest

E.G.M. reports an unrestricted research grant from Allergy Therapeutics Ibérica supporting this project; compensation was limited to fair market value for time and work related to the Delphi process. E.G.M. has received consulting fees from Diater Laboratorio de Diagnóstico y Aplicaciones Terapéuticas and LETI Pharma; honoraria for lectures or presentations from Allergy Therapeutics Ibérica, Diater Laboratorio de Diagnóstico y Aplicaciones Terapéuticas, Inmunotek, Stallergenes Greer, ALK‐Abelló, LETI Pharma, and ROXALL Group; and support for attending meetings from Allergy Therapeutics Ibérica, Diater Laboratorio de Diagnóstico y Aplicaciones Terapéuticas, Inmunotek, LETI Pharma, and ROXALL Group. EGM is the Chair of the Immunotherapy Committee of the SEAIC. J.M.B.M. reports an unrestricted research grant from Allergy Therapeutics Ibérica supporting this project; compensation was limited to fair market value for time and work related to the Delphi process. J.M.B.M. has received consulting fees from LETI Pharma and Asac Pharmaceutical Immunology; honoraria for lectures or presentations from LETI Pharma, Asac Pharmaceutical Immunology, Diater Laboratorio de Diagnóstico y Aplicaciones Terapéuticas, Stallergenes Greer, ALK‐Abelló and ROXALL Group; and support for attending meetings from LETI Pharma, Diater Laboratorio de Diagnóstico y Aplicaciones Terapéuticas and ROXALL Group. A.I.T.P. reports an unrestricted research grant from Allergy Therapeutics Ibérica supporting this project; compensation was limited to fair market value for time and work related to the Delphi process. A.I.T.P. reports receiving consultancy or speaking fees from: Allergy Therapeutics Ibérica, ALK‐Abelló, Diater Laboratorio de Diagnóstico y Aplicaciones Terapéuticas, Inmunotek, InnoUp Farma, ITAI Pharma, LETI Pharma, Probelte Pharma and ROXALL Group and participated in research projects sponsored by: Allergy Therapeutics Ibérica, Diater Laboratorio de Diagnóstico y Aplicaciones Terapéuticas, Inmunotek, InnoUp Farma, GlaxoSmithKline and LETI Pharma. M.G.C. reports an unrestricted research grant from Allergy Therapeutics Ibérica supporting this project; compensation was limited to fair market value for time and work related to the Delphi process. M.G.C. has received honoraria for lectures or presentations from Sanofi, GlaxoSmithKline, AstraZeneca; and support for attending meetings from Stallergenes Greer, Diater Laboratorio de Diagnóstico y Aplicaciones Terapéuticas, LETI Pharma, ROXALL Group, GlaxoSmithKline, Allergy Therapeutics Ibérica. A.I.D. reports an unrestricted research grant from Allergy Therapeutics Ibérica supporting this project; compensation was limited to fair market value for time and work related to the Delphi process. A.I.D. has received payment or honoraria for lectures, presentations, speakers' bureaus, manuscript writing or educational events from GlaxoSmithKline, Viatris Pharmaceuticals, Sanofi, Lofarma Ibérica, Novartis Farmacéutica, Laboratorios Menarini, Organon Salud, Uriach, Inmunotek, LETI Pharma, Diater Laboratorio de Diagnóstico y Aplicaciones Terapéuticas, AstraZeneca and Optinose. A.I.D. is the Vice‐Chair of the Rhinitis and Allergic Conjunctivitis Committee of the SEAIC. M.J.C.M. reports an unrestricted research grant from Allergy Therapeutics Ibérica supporting this project; compensation was limited to fair market value for time and work related to the Delphi process. C.C.B. reports an unrestricted research grant from Allergy Therapeutics Ibérica supporting this project; compensation was limited to fair market value for time and work related to the Delphi process. Participation on an Advisory Board supported by Stallergenes Greer and support for attending meetings from Stallergenes Greer, Diater Laboratorio de Diagnóstico y Aplicaciones Terapéuticas, LETI Pharma, Allergy Therapeutics Ibérica, Allergopharma, Probelte Pharma, Faes Farma. L.D.C. reports an unrestricted research grant from Allergy Therapeutics Ibérica supporting this project; compensation was limited to fair market value for time and work related to the Delphi process. L.D.C. has received payment or honoraria for presentations, support for manuscript writing or educational events; and support for attending meetings from Chiesi, LETI Pharma and Allergy Therapeutics Ibérica. A.E.M. reports an unrestricted research grant from Allergy Therapeutics Ibérica supporting this project; compensation was limited to fair market value for time and work related to the Delphi process. A.E.M. also reports receiving payment or honoraria for lectures, presentations, speakers' bureaus, manuscript writing or educational events from Allergy Therapeutics Ibérica, Stallergenes Greer, Diater Laboratorio de Diagnóstico y Aplicaciones Terapéuticas, Novartis Farmacéutica, LETI Pharma, Orion Pharma, Laboratorios Menarini, Laboratorios Gebro Pharma and support for attending meetings from Inmunotek and LETI Pharma. IEG reports an unrestricted research grant from Allergy Therapeutics Ibérica supporting this project; compensation was limited to fair market value for time and work related to the Delphi process. I.E.G. has received payment or honoraria for presentations, support for manuscript writing or educational events from Diater Laboratorio de Diagnóstico y Aplicaciones Terapéuticas, LETI Pharma, AstraZeneca; and support for attending meetings from LETI Pharma, Inmunotek, Diater Laboratorio de Diagnóstico y Aplicaciones Terapéuticas and ROXALL Group. A.G.S. reports an unrestricted research grant from Allergy Therapeutics Ibérica supporting this project; compensation was limited to fair market value for time and work related to the Delphi process. A.G.S. has received payment or honoraria for presentations, support for manuscript writing or educational events; and support for attending meetings from Chiesi, GlaxoSmithKline, Lofarma Ibérica, Novartis Farmacéutica, Laboratorios Menarini, Inmunotek, ROXALL Group, LETI Pharma and Diater Laboratorio de Diagnóstico y Aplicaciones Terapéuticas. A.J.G. reports an unrestricted research grant from Allergy Therapeutics Ibérica supporting this project; compensation was limited to fair market value for time and work related to the Delphi process.

## Supporting information


Supporting Information S1

Supporting Information S2

**Table S1:** Methodological plan guiding the Delphi consensus process.

**Table S2:** DELPHISTAR reporting checklist for Delphi studies.

## Data Availability

The data that support the findings of this study are available from the corresponding author upon reasonable request.
