# Supplementary material for: Criteria for Control and Remission of Respiratory Allergic Disease With Allergen Immunotherapy: A Delphi Consensus
Source: Clin Transl Allergy. 2026 Aug 2;16(8):e70191. doi: 10.1002/clt2.70191 (PMC13429802; doi:10.1002/clt2.70191)
Supplement: Supplementary file 1 — Supporting Information S1 [file CLT2-16-e70191-s004.docx]

**This is an English translation of the original Spanish questionnaire used in the survey**

This document presents the questions that were asked to the panellists in both waves of the Delphi. Questions that were only answered in wave 2 are indicated in blue .

# Delphi Questionnaire

## Section 1: Participant Profile

Before you begin answering the Delphi questionnaire, it would be very helpful if you could answer these questions related **to your profile and the characterisation of your usual clinical practice** in relation to patients with **allergic rhinitis, allergic conjunctivitis and/or allergic asthma.**

**1.1 What is your position at the centre where you treat most of your patients with allergic diseases?**

Please select only one answer

- Head of department
- Head of section
- Doctor
- Resident [END]

**1.2 How many years have you been responsible for treating patients with ALLERGIC DISEASES (allergic rhinitis, allergic conjunctivitis and/or allergic asthma) and prescribing SPECIFIC ALLERGEN IMMUNOTHERAPY (SAIT) as treatment? (count from the end of your specialisation/residency)***

Please select only one answer

- < 5 years [END]
- 5 to 10 years (not counting MIR)
- 11 to 15 years (not counting MIR)
- 16 to 20 years (not counting MIR)
- > 20 years (not counting MIR)

**1.3 Indicate your main speciality***

Please select only one answer

- Allergology
- Other: _________ [END]

**1.4 In your regular clinical practice, thinking about the last month or so, how many patients on average do you usually treat for allergic diseases (allergic rhinitis, allergic conjunctivitis and/or allergic asthma)? (think about patients, not visits) ***

Please enter a numerical answer

__ Number of patients with allergic diseases in the last month (regardless of whether they have allergic rhinitis, allergic conjunctivitis and/or allergic asthma)

**1.5 Of the patients mentioned above, what percentage are adult and paediatric patients, respectively? (think in terms of patients, not visits) ***

Please provide the percentage of adult and paediatric patients separately.

___% adult patients with allergic disease (regardless of whether they suffer from allergic rhinitis, allergic conjunctivitis and/or allergic asthma)

___% paediatric patients with allergic disease (regardless of whether they have allergic rhinitis, allergic conjunctivitis and/or allergic asthma)

**1.6 Have you contributed as a researcher to clinical trials, observational studies, real-world studies, or other studies related to allergic disease or SAIT in the last 5 years?***

Please select the appropriate answers

- Yes, in relation to allergic disease (opens question P1.6a)
- Yes, in relation to SAIT (opens question P1.6b)
- No

1.6a. Please specify number (in relation to allergic disease), n=______ (open-ended response if they answer "Yes" to the previous question)

1.6b. Please specify number (in relation to SAIT), n=______ (open-ended response if they answer "Yes" to the previous question)

**1.7 Have you contributed as an author to publications related to allergic disease or SAIT in the last 5 years? ***

Please select only one answer

- Yes, in relation to allergic disease (opens question S1_7_a)
- Yes, in relation to SAIT (opens question S1_7_b)
- No

1.7a. Please specify number (in relation to allergic disease), n=______ (open response if they answer 'Yes' to the previous question)

1.7b. Please specify number (related to SAIT), n=______(open-ended response if they answered "Yes" to the previous question)

**1.8 Do you carry out teaching activities related to allergic disease or SAIT? ***

Please select only one answer

- Yes
- No

**1.9 Are you or have you been a member of the SEAIC Immunotherapy Committee? ***

Please select only one answer

- Yes
- No

**1.10 Please indicate the type of centre where you treat your patients with allergic diseases: ***

Please select only one answer

- In a public centre
- In a private centre
- Both (public and private)

**1.11 The centre where you treat most of your patients belongs to: …***

*^i^https://www.sanidad.gob.es/estadEstudios/estadisticas/docs/NormaGRD2008/CLASIFICACIONHOSPITALESCLUSTER.pdf*

Please select only one answer

- GROUP 1: Regional hospital, with less than 150 beds on average, not highly complex.
- GROUP 2: Basic general hospital, medium size with fewer than 200 beds, and somewhat higher complexity.
- GROUP 3: Area hospital, medium size with around 500 beds. Medium complexity.
- GROUP 4: Large hospital group, but more heterogeneous in terms of staffing, size and activity. High teaching intensity, high complexity.
- GROUP 5: Hospital with significant structural weight and high activity. Full range of services. Includes large complexes.

**1.12 Is there an immunotherapy unit at the centre where you treat your patients? ***

Please select only one answer

- Yes
- No

**1.13** Finally, please **indicate your gender: ***

Please select only one answer

- Male
- Female
- I prefer not to answer

**1.14 Indicate your year of birth: ***

Numerical response

_ _ _

**1.15 Indicate the main autonomous community where you currently work: ***

Please select only one answer

- Andalusia
- Aragon
- Canary Islands
- Cantabria
- Castile-La Mancha
- Castile and León
- Catalonia
- Ceuta
- Community of Madrid
- Formal Community of Navarre
- Valencian Community
- Extremadura
- Galicia
- Balearic Islands
- La Rioja
- Melilla
- Basque Country
- Principality of Asturias
- Region of Murcia

## Section 2. Allergic rhinitis

### 2.1 Criteria for good control of allergic rhinitis

In this section, we will ask you about **criteria for controlling allergic rhinitis** in patients undergoing treatment with SAIT, regardless of whether they are also taking basic pharmacological treatment. These criteria refer to the allergen(s) for which the patient is undergoing treatment.

P2.1.1 **Thinking about patients undergoing treatment with SAIT, regardless of concomitant basic pharmacological treatment and referring to the allergen(s) for which the patient is undergoing treatment, indicate your degree of agreement that the following items constitute the definition of GOOD CONTROL of ALLERGIC RHINITIS *(where 1 is strongly disagree and 9 is strongly agree)***

| Indicators for defining GOOD CONTROL of ALLERGIC RHINITIS. | **Strongly disagree** | | |  | | | | | | | **Strongly agree** |  |
| --- | --- | --- | --- | --- | --- | --- | --- | --- | --- | --- | --- | --- |
| 1. RCAT questionnaire score (6-30) between 23-30.*   *^i^allergyasthmanetwork.org/images/Misc/rhinitis_control_assessment_test_RCAT.pdf / RCAT (Rhinitis Control Assessment Test)* | | 1 | 2 | | 3 | 4 | 5 | 6 | 7 | 8 | 9 | |
| 1. Absence of daytime symptoms of allergic rhinitis (nasal itching, nasal congestion, rhinorrhoea, sneezing).* | | 1 | 2 | | 3 | 4 | 5 | 6 | 7 | 8 | 9 | |
| 1. Presence of daytime symptoms of allergic rhinitis less than 2 days per month (nasal itching, nasal congestion, runny nose, sneezing).*   [IF THE PANELLIST INDICATES 1, 2 OR 3, THE FOLLOWING QUESTION WILL APPEAR]: You have indicated that you disagree with the previous statement. If your disagreement is related to frequency, what would be the appropriate frequency for you? | | 1 | 2 | | 3 | 4 | 5 | 6 | 7 | 8 | 9 | |
| 1. Absence of nocturnal symptoms of allergic rhinitis (nasal pruritus, nasal congestion, rhinorrhoea, sneezing). * | | 1 | 2 | | 3 | 4 | 5 | 6 | 7 | 8 | 9 | |
| 1. Nasal symptoms measured by VAS (0-10) ≤3 cm (nasal itching, nasal congestion, rhinorrhoea, sneezing).*   *^i^ VAS (visual analogue scale)* | | 1 | 2 | | 3 | 4 | 5 | 6 | 7 | 8 | 9 | |
| 1. Score on the RQLQ quality of life questionnaire (0-6) of 0-2.   ^i^ RQLQ (Rhinitis Quality of Life Questionnaire) | | 1 | 2 | | 3 | 4 | 5 | 6 | 7 | 8 | 9 | |
| 1. No limitation of the patient's daily activities (e.g. usual tasks at work, school or home).* | | 1 | 2 | | 3 | 4 | 5 | 6 | 7 | 8 | 9 | |
| 1. Absence of symptomatic medication use. * | | 1 | 2 | | 3 | 4 | 5 | 6 | 7 | 8 | 9 | |
| 1. Use of symptomatic medication less than 2 days per month. *   [IF THE PANELLIST INDICATES 1, 2 OR 3, THE FOLLOWING QUESTION WILL APPEAR]: You have indicated that you disagree with the previous statement. If your disagreement is related to frequency, what would be the appropriate frequency for you? | | 1 | 2 | | 3 | 4 | 5 | 6 | 7 | 8 | 9 | |
| 1. Nasal function/permeability indicated by a PFIN value > 80% compared to baseline. *   *^i^ PFIN (peak nasal inspiratory flow)* | | 1 | 2 | | 3 | 4 | 5 | 6 | 7 | 8 | 9 | |
| 1. Absence of exacerbations. *   ^i^ *Considering exacerbation as the reappearance of symptoms affecting quality of life and requiring symptomatic treatment after exposure to the allergen to which the patient is sensitised.* | | 1 | 2 | | 3 | 4 | 5 | 6 | 7 | 8 | 9 | |

P2.1.2 **Thinking about patients undergoing treatment with SAIT, regardless of concomitant basic pharmacological treatment and referring to the allergen(s) for which the patient is undergoing treatment, indicate your degree of agreement that the following item constitutes the definition of GOOD CONTROL of ALLERGIC RHINITIS *(where 1 is strongly disagree and 9 is strongly agree)***

| Number of indicators to define GOOD CONTROL of ALLERGIC RHINITIS. | **Strongly disagree** | |  | | | | | | | **Strongly agree** | |
| --- | --- | --- | --- | --- | --- | --- | --- | --- | --- | --- | --- |
| 1. Compliance with ALL of the above criteria for good control is necessary to define allergic rhinitis as well controlled*. | 1 | 2 | | 3 | 4 | 5 | 6 | 7 | 8 | | 9 |

Questions included in wave 2

**Considering the applicability for use in clinical practice, please express your level of agreement with the following definition of good control of allergic rhinitis:**

| Indicator for defining GOOD CONTROL of ALLERGIC RHINITIS. | **Strongly disagree** | |  | | | | | | | **Strongly agree** | |
| --- | --- | --- | --- | --- | --- | --- | --- | --- | --- | --- | --- |
| To define GOOD CONTROL of ALLERGIC RHINITIS, all clinical criteria must be met:   - **Absence of daytime symptoms** of allergic rhinitis (nasal itching, nasal congestion, rhinorrhoea, sneezing) or presence of these symptoms **less than 2 days per month.** - **Absence of nocturnal symptoms** of allergic rhinitis (nasal itching, nasal congestion, rhinorrhoea, sneezing). - **No limitation of** the patient's daily **activities** (e.g. usual tasks at work, school or home). - **Absence of** symptomatic **medication use** or use for **less than 2 days per month.** - **Absence of exacerbations.**   And at least one of the following:   - **RCAT** questionnaire score **(6-30) between 23-30.** - Nasal symptoms measured by **VAS (0-10) ≤3 cm** (nasal itching, nasal congestion, rhinorrhoea, sneezing). - **RQLQ** quality of life questionnaire score **(0-6) of 0-2.** - Nasal function/permeability indicated by a **PFIN** value **> 80%** compared to baseline. | 1 | 2 | | 3 | 4 | 5 | 6 | 7 | 8 | | 9 |

### Partial control criteria for allergic rhinitis

P2.2.1 **Thinking about patients undergoing treatment with SAIT, regardless of concomitant basic pharmacological treatment and referring to the allergen(s) for which the patient is undergoing treatment, indicate your degree of agreement that the following items constitute the definition of PARTIAL CONTROL of ALLERGIC RHINITIS *(where 1 is strongly disagree and 9 is strongly agree)***

| Indicators for defining PARTIAL CONTROL of ALLERGIC RHINITIS. | **Strongly disagree** | |  | | | | | | | **Strongly agree** | |
| --- | --- | --- | --- | --- | --- | --- | --- | --- | --- | --- | --- |
| 1. RCAT questionnaire score (6-30) <23. *   *^i^allergyasthmanetwork.org/images/Misc/rhinitis_control_assessment_test_RCAT.pdf / RCAT (Rhinitis Control Assessment Test)* |  |  | |  |  |  |  |  |  | |  |
| 1. Presence of daytime symptoms of allergic rhinitis between 2 and 7 days per month (nasal itching, nasal congestion, rhinorrhoea, sneezing). *   [IF THE PANELLIST INDICATES 1, 2 OR 3, THE FOLLOWING QUESTION WILL APPEAR]: You have indicated that you disagree with the previous statement. If your disagreement is related to frequency, what would be the appropriate frequency for you? | 1 | 2 | | 3 | 4 | 5 | 6 | 7 | 8 | | 9 |
| 1. Are there any nocturnal symptoms of allergic rhinitis (nasal itching, nasal congestion, rhinorrhoea, sneezing)? * | 1 | 2 | | 3 | 4 | 5 | 6 | 7 | 8 | | 9 |
| 1. Nasal symptoms measured by VAS (0-10) from 4-7 cm (nasal itching, nasal congestion, rhinorrhoea, sneezing).   *^I^ VAS (visual analogue scale)* | 1 | 2 | | 3 | 4 | 5 | 6 | 7 | 8 | | 9 |
| 1. Score on the RQLQ quality of life questionnaire (0-6) of 3-4.   ^i^ RQLQ (Rhinitis Quality of Life Questionnaire) | 1 | 2 | | 3 | 4 | 5 | 6 | 7 | 8 | | 9 |
| 1. Are there any limitations on the patient's daily activities (e.g., usual tasks at work, school, or home)?*. | 1 | 2 | | 3 | 4 | 5 | 6 | 7 | 8 | | 9 |
| 1. Use of symptomatic medication between 2 and 7 days per month. *   [IF THE PANELLIST INDICATES 1, 2 OR 3, THE FOLLOWING QUESTION WILL APPEAR]: You have indicated that you disagree with the previous statement. If your disagreement is related to frequency, what would be the appropriate frequency for you? | 1 | 2 | | 3 | 4 | 5 | 6 | 7 | 8 | | 9 |
| 1. Nasal function/permeability indicated by a PFIN value > 80% compared to baseline. *   *^i^ PFIN (peak nasal inspiratory flow)* | 1 | 2 | | 3 | 4 | 5 | 6 | 7 | 8 | | 9 |
| 1. Having one or more exacerbations per year.*   *^i^ Considering exacerbation as the reappearance of symptoms affecting quality of life and requiring symptomatic treatment after exposure to the allergen to which the patient is allergic.* | 1 | 2 | | 3 | 4 | 5 | 6 | 7 | 8 | | 9 |

Questions included in wave 2

**As you can see from the graph above, no consensus was reached on the following criterion. Please indicate your level of agreement with the following criterion being part of the definition of PARTIAL CONTROL of ALLERGIC RHINITIS.**

| Indicator for defining PARTIAL CONTROL of ALLERGIC RHINITIS. | **Strongly disagree** | |  | | | | | | | **Strongly agree** | |
| --- | --- | --- | --- | --- | --- | --- | --- | --- | --- | --- | --- |
| 1. RCAT questionnaire score (6-30) <23. *   *^i^allergyasthmanetwork.org/images/Misc/rhinitis_control_assessment_test_RCAT.pdf / RCAT (Rhinitis Control Assessment Test)* | 1 | 2 | | 3 | 4 | 5 | 6 | 7 | 8 | | 9 |

P2.2.2 **Thinking about patients undergoing treatment with SAIT, regardless of concomitant basic pharmacological treatment and referring to the allergen(s) for which the patient is undergoing treatment, indicate your degree of agreement that the following item constitutes the definition of PARTIAL CONTROL of ALLERGIC RHINITIS *(where 1 is strongly disagree and 9 is strongly agree)***

| Number of indicators to define PARTIAL CONTROL of ALLERGIC RHINITIS | **Strongly disagree** | |  | | | | | | | **Strongly agree** | |
| --- | --- | --- | --- | --- | --- | --- | --- | --- | --- | --- | --- |
| 1. Fulfilling LESS THAN 3 of the above criteria for partial control is necessary to define allergic rhinitis as partially controlled. * | 1 | 2 | | 3 | 4 | 5 | 6 | 7 | 8 | | 9 |

### 2.3 Criteria for poor control of allergic rhinitis

P2.3.1 **Considering patients undergoing treatment with SAIT, regardless of concomitant basic pharmacological treatment and referring to the allergen(s) for which the patient is undergoing treatment, indicate your degree of agreement that the following items constitute the definition of POOR CONTROL of ALLERGIC RHINITIS *(where 1 is strongly disagree and 9 is strongly agree)***

| Indicators for defining POOR CONTROL of ALLERGIC RHINITIS. | **Strongly disagree** | |  | | | | | | | **Strongly agree** | |
| --- | --- | --- | --- | --- | --- | --- | --- | --- | --- | --- | --- |
| 1. Any situation not covered by the criteria for good control or partial control should be considered poor control of allergic rhinitis. * | 1 | 2 | | 3 | 4 | 5 | 6 | 7 | 8 | | 9 |
| 1. Three or more characteristics of partially controlled rhinitis must be present to define allergic rhinitis as poorly controlled.* | 1 | 2 | | 3 | 4 | 5 | 6 | 7 | 8 | | 9 |
| 1. Nasal symptoms measured by VAS (0-10) >7cm (nasal itching, nasal congestion, rhinorrhoea, sneezing)   *^i^ VAS (visual analogue scale)* | 1 | 2 | | 3 | 4 | 5 | 6 | 7 | 8 | | 9 |
| 1. Score on the RQLQ quality of life questionnaire (0-6) of 5-6.   ^i^ RQLQ (Rhinitis Quality of Life Questionnaire) | 1 | 2 | | 3 | 4 | 5 | 6 | 7 | 8 | | 9 |

### 2.4 Criteria for clinical remission of allergic rhinitis

In this section, we will ask you about **the criteria for clinical remission of allergic rhinitis** in patients undergoing treatment with SAIT. Please note that these criteria refer to the allergen(s) for which the patient is undergoing treatment and that, in order to assess remission, the patient must not require concomitant basic pharmacological treatment. Think in general terms and without taking into account the duration of remission, as we will address that aspect in *Section 5: General Concepts*.

P2.4.1 **Thinking about patients undergoing treatment with SAIT and referring to the allergen(s) for which the patient is undergoing treatment, indicate your degree of agreement that the following items constitute the definition of CLINICAL REMISSION of ALLERGIC RHINITIS *(where 1 is strongly disagree and 9 is strongly agree)*.**

| Indicators for defining CLINICAL REMISSION of ALLERGIC RHINITIS. | **Strongly disagree** | |  | | | | | | | **Strongly agree** | |
| --- | --- | --- | --- | --- | --- | --- | --- | --- | --- | --- | --- |
| 1. Absence of nasal symptoms (nasal itching, nasal congestion, rhinorrhoea, sneezing). * | 1 | 2 | | 3 | 4 | 5 | 6 | 7 | 8 | | 9 |
| 1. No need for symptomatic treatment. * | 1 | 2 | | 3 | 4 | 5 | 6 | 7 | 8 | | 9 |
| 1. Nasal function indicated by a PFIN value > 80% compared to personal best. *   *^i^PFIN (peak nasal inspiratory flow)* | 1 | 2 | | 3 | 4 | 5 | 6 | 7 | 8 | | 9 |

## Section 3: Allergic conjunctivitis

### 3.1 Criteria for good control of allergic conjunctivitis

In this section, we will ask you about **criteria for controlling allergic conjunctivitis** in patients undergoing treatment with SAIT, regardless of whether they are also taking basic pharmacological treatment. These criteria refer to the allergen(s) for which the patient is undergoing treatment.

P3.1.1 **Thinking about patients undergoing treatment with SAIT, regardless of concomitant basic pharmacological treatment and referring to the allergen(s) for which the patient is undergoing treatment, indicate your degree of agreement that the following items constitute the definition of GOOD CONTROL of ALLERGIC CONJUNCTIVITIS *(where 1 is strongly disagree and 9 is strongly agree)***

| Indicators for defining GOOD CONTROL of ALLERGIC CONJUNCTIVITIS. | **Strongly disagree** | |  | | | | | | | **Strongly agree** | |
| --- | --- | --- | --- | --- | --- | --- | --- | --- | --- | --- | --- |
| 1. Absence of ocular symptoms (itching, tearing, conjunctival erythema) | 1 | 2 | | 3 | 4 | 5 | 6 | 7 | 8 | | 9 |
| 1. Presence of eye symptoms less than 2 days per month (itching, tearing, visual discomfort). *   [IF THE PANELLIST INDICATES 1, 2 OR 3, THE FOLLOWING QUESTION WILL APPEAR]: You have indicated that you disagree with the previous statement. If your disagreement is related to frequency, what would be the appropriate frequency for you? | 1 | 2 | | 3 | 4 | 5 | 6 | 7 | 8 | | 9 |
| 1. Ocular symptoms measured by VAS (0-10) ≤3 cm.*   *^i^ VAS (visual analogue scale)* | 1 | 2 | | 3 | 4 | 5 | 6 | 7 | 8 | | 9 |
| 1. Score on the RQLQ quality of life questionnaire (0-6) of 0-2.   ^i^ RQLQ (Rhinitis Quality of Life Questionnaire) | 1 | 2 | | 3 | 4 | 5 | 6 | 7 | 8 | | 9 |
| 1. Level of hyperaemia assessed on the *Efron* scale (0-4) with a score of 0-1. *   *^i^*  *https://www.jiaci.org/summary/vol25-issue2-num1203* | 1 | 2 | | 3 | 4 | 5 | 6 | 7 | 8 | | 9 |
| 1. No use of symptomatic medication. * | 1 | 2 | | 3 | 4 | 5 | 6 | 7 | 8 | | 9 |
| 1. Use of symptomatic medication less than 2 days per month. *   [IF THE PANELLIST INDICATES 1, 2 OR 3, THE FOLLOWING QUESTION WILL APPEAR]: You have indicated that you disagree with the previous statement. If your disagreement is related to frequency, what would be the appropriate frequency for you? | 1 | 2 | | 3 | 4 | 5 | 6 | 7 | 8 | | 9 |

P3.1.2 **Thinking about patients undergoing treatment with SAIT, regardless of concomitant basic pharmacological treatment and referring to the allergen(s) for which the patient is undergoing treatment, indicate your degree of agreement or disagreement with the following item constituting the definition of GOOD CONTROL of ALLERGIC CONJUNCTIVITIS *(where 1 is strongly disagree and 9 is strongly agree)***

| Number of indicators to define GOOD CONTROL of ALLERGIC CONJUNCTIVITIS | **Strongly disagree** | |  | | | | | | | **Strongly agree** | |
| --- | --- | --- | --- | --- | --- | --- | --- | --- | --- | --- | --- |
| 1. Compliance with ALL of the above criteria for good control is necessary to define allergic conjunctivitis as well controlled*. | 1 | 2 | | 3 | 4 | 5 | 6 | 7 | 8 | | 9 |

Questions included in wave 2

**Considering the applicability for use in clinical practice, please express your level of agreement with the following definition of good control of allergic conjunctivitis:**

| Indicator for defining GOOD CONTROL of ALLERGIC CONJUNCTIVITIS. | **Strongly disagree** | |  | | | | | | | **Strongly agree** | |
| --- | --- | --- | --- | --- | --- | --- | --- | --- | --- | --- | --- |
| To define GOOD CONTROL of ALLERGIC CONJUNCTIVITIS, all clinical criteria must be met:   - **Absence of ocular symptoms** (itching, tearing, visual discomfort) or presence of these symptoms **for less than 2 days per month.** - **Absence** of **symptomatic medication** use or use **less than 2 days per month.**   And at least one of the following:   - Ocular symptoms measured by **VAS (0-10) ≤3 cm.** - **RQLQ** quality of life questionnaire score **(0-6) of 0-2.** - Level of hyperaemia assessed on the **Efron** scale **(0-4) with a score of 0-1**. | 1 | 2 | | 3 | 4 | 5 | 6 | 7 | 8 | | 9 |

### 3.2 Criteria for partial control of allergic conjunctivitis

P3.2.1 **Considering patients undergoing treatment with SAIT, regardless of concomitant basic pharmacological treatment and referring to the allergen(s) for which the patient is undergoing treatment, indicate your degree of agreement that the following items constitute the definition of PARTIAL CONTROL of ALLERGIC CONJUNCTIVITIS *(where 1 is strongly disagree and 9 is strongly agree)***

| Indicators for defining PARTIAL CONTROL of ALLERGIC CONJUNCTIVITIS. | **Strongly disagree** | |  | | | | | | | **Strongly agree** | |
| --- | --- | --- | --- | --- | --- | --- | --- | --- | --- | --- | --- |
| 1. Presence of eye symptoms between 2 and 7 days per month (itching, tearing, visual discomfort). *   [IF THE PANELLIST INDICATES 1, 2 OR 3, THE FOLLOWING QUESTION WILL APPEAR]: You have indicated that you disagree with the previous statement. If your disagreement is related to frequency, what would be the appropriate frequency for you? | 1 | 2 | | 3 | 4 | 5 | 6 | 7 | 8 | | 9 |
| 1. Ocular symptoms measured by VAS (0-10) of 4-7 cm.   *^I^ VAS (visual analogue scale)* | 1 | 2 | | 3 | 4 | 5 | 6 | 7 | 8 | | 9 |
| 1. Score on the RQLQ quality of life questionnaire (0-6) of 3-4.   ^i^ RQLQ (Rhinitis Quality of Life Questionnaire) | 1 | 2 | | 3 | 4 | 5 | 6 | 7 | 8 | | 9 |
| 1. Level of hyperaemia assessed on the *Efron* scale (0-4) with a score of 2. *   *^i^*  *https://www.jiaci.org/summary/vol25-issue2-num1203* | 1 | 2 | | 3 | 4 | 5 | 6 | 7 | 8 | | 9 |
| 1. Use of symptomatic medication between 2 and 7 days per month. *   [IF THE PANELLIST INDICATES 1, 2 OR 3, THE FOLLOWING QUESTION WILL APPEAR]: You have indicated that you disagree with the previous statement. If your disagreement is related to frequency, what would be the appropriate frequency for you? | 1 | 2 | | 3 | 4 | 5 | 6 | 7 | 8 | | 9 |

P3.2.2 **Thinking about patients undergoing treatment with SAIT, regardless of concomitant basic pharmacological treatment and referring to the allergen(s) for which the patient is undergoing treatment, indicate your degree of agreement that the following item constitutes the definition of PARTIAL CONTROL of ALLERGIC CONJUNCTIVITIS *(where 1 is strongly disagree and 9 is strongly agree)***

| Number of indicators to define PARTIAL CONTROL of ALLERGIC RHINITIS | **Strongly disagree** | |  | | | | | | | **Strongly agree** | |
| --- | --- | --- | --- | --- | --- | --- | --- | --- | --- | --- | --- |
| 1. Fulfilling LESS THAN 3 of the above criteria for partial control is necessary to define allergic conjunctivitis as partially controlled. * | 1 | 2 | | 3 | 4 | 5 | 6 | 7 | 8 | | 9 |

### 3.3 Criteria for poor control of allergic conjunctivitis

P3.3.1 **Considering patients undergoing treatment with SAIT, regardless of concomitant basic pharmacological treatment and referring to the allergen(s) for which the patient is undergoing treatment, indicate your degree of agreement that the following items constitute the definition of POOR CONTROL of ALLERGIC CONJUNCTIVITIS *(where 1 is strongly disagree and 9 is strongly agree)***

| Indicators for defining POOR CONTROL of ALLERGIC CONJUNCTIVITIS. | **Strongly disagree** | |  | | | | | | | **Strongly agree** | |
| --- | --- | --- | --- | --- | --- | --- | --- | --- | --- | --- | --- |
| 1. Any situation not covered by the criteria for good control or partial control should be considered poor control of allergic conjunctivitis. * | 1 | 2 | | 3 | 4 | 5 | 6 | 7 | 8 | | 9 |
| 1. Three or more characteristics of partially controlled conjunctivitis must be present to define allergic rhinitis as poorly controlled.* | 1 | 2 | | 3 | 4 | 5 | 6 | 7 | 8 | | 9 |
| 1. Ocular symptoms measured by VAS (0-10) >7 cm.*   *^i^ VAS (visual analogue scale)* | 1 | 2 | | 3 | 4 | 5 | 6 | 7 | 8 | | 9 |
| 1. Score on the RQLQ quality of life questionnaire (0-6) of 5-6.   ^i^ RQLQ (Rhinitis Quality of Life Questionnaire) | 1 | 2 | | 3 | 4 | 5 | 6 | 7 | 8 | | 9 |
| 1. Level of hyperaemia assessed on the *Efron* scale (0-4) with a score of 3-4. *   *^i^*  *https://www.jiaci.org/summary/vol25-issue2-num1203* | 1 | 2 | | 3 | 4 | 5 | 6 | 7 | 8 | | 9 |

### 3.4 Criteria for clinical remission of allergic conjunctivitis

In this section, we will ask you about **the criteria for clinical remission of allergic conjunctivitis** in patients undergoing treatment with SAIT. Please note that these criteria refer to the allergen(s) for which the patient is undergoing treatment and that, in order to assess remission, the patient must not require concomitant basic pharmacological treatment. Think in general terms and without taking into account the duration of remission, as we will address that aspect in *Section 5: General Concepts*.

P3.4.1 **Thinking about patients undergoing treatment with SAIT and referring to the allergen(s) for which the patient is undergoing treatment, indicate your degree of agreement that the following items constitute the definition of CLINICAL REMISSION of ALLERGIC CONJUNCTIVITIS *(where 1 is strongly disagree and 9 is strongly agree)***

| Indicators for defining CLINICAL REMISSION of ALLERGIC CONJUNCTIVITIS. | **Strongly disagree** | |  | | | | | | | **Strongly agree** | |
| --- | --- | --- | --- | --- | --- | --- | --- | --- | --- | --- | --- |
| 1. Absence of ocular symptoms and signs. * | 1 | 2 | | 3 | 4 | 5 | 6 | 7 | 8 | | 9 |
| 1. No need for symptomatic treatment. * | 1 | 2 | | 3 | 4 | 5 | 6 | 7 | 8 | | 9 |

## Section 4: Allergic asthma

The aim of this section is to reach a consensus on whether it is appropriate to apply the current criteria **defining asthma control and remission to allergic asthma** in patients undergoing **treatment with SAIT**, regardless of concomitant basic pharmacological treatment. These criteria refer to the allergen(s) for which the patient is undergoing treatment.

### 4.1 Criteria for good control of allergic asthma

P4.1.1 **Considering patients undergoing treatment with SAIT, regardless of concomitant basic pharmacological treatment and referring to the allergen(s) for which the patient is undergoing treatment, indicate your degree of agreement that the following items constitute the definition of GOOD CONTROL of asthma, as set out in the GEMA 5.4 guideline, applicable to ALLERGIC ASTHMA** ***(where 1 = strongly disagree and 9 = strongly agree)***

| Indicators for defining GOOD CONTROL of ALLERGIC ASTHMA | **Strongly disagree** |  | | | | | | | **Strongly agree** |
| --- | --- | --- | --- | --- | --- | --- | --- | --- | --- |
| 1. *All of the following criteria must be met:*  - No daytime symptoms or ≤ 2 days per month - No limitation of activities - No night-time symptoms - Use of rescue medication: none or ≤ 2 days per month - Lung function:   - 1. FEV1 ≥ 80% of predicted     2. PEF ≥ 80% of personal best - No exacerbations. | 1 | 2 | 3 | 4 | 5 | 6 | 7 | 8 | 9 |

[IF THE PANELLIST INDICATES 1, 2 OR 3, THE FOLLOWING QUESTION WILL APPEAR]: You have indicated that you disagree that the criteria for good control based on the GEMA 5.4 guideline **are applicable to allergic asthma**. Please could you explain **in detail your reasons** for disagreeing with this definition?

_________________________________ (open question)

P4.1.2 **Thinking about patients undergoing treatment with SAIT, regardless of concomitant basic pharmacological treatment and referring to the allergen(s) for which the patient is undergoing treatment, indicate your degree of agreement that the following items (referring to the use of PROMs) also constitute the definition of GOOD CONTROL of asthma *(where 1 = strongly disagree and 9 = strongly agree)***

| Additional indicators to define GOOD CONTROL of ALLERGIC ASTHMA | **Strongly disagree** | |  | | | | | | | **Strongly agree** | |
| --- | --- | --- | --- | --- | --- | --- | --- | --- | --- | --- | --- |
| 1. Allergic asthma symptoms measured with a score of ≤3 cm on the VAS scale (0-10).   *^i^VAS (visual analogue scale)* | 1 | 2 | | 3 | 4 | 5 | 6 | 7 | 8 | | 9 |
| 1. ACT questionnaire score (5-25) ≥ 20.   *^i^https://www.svnpar.es/wp-content/uploads/2018/11/Test_ACT.pdf/ACT (Asthma Control Test)* | 1 | 2 | | 3 | 4 | 5 | 6 | 7 | 8 | | 9 |
| 1. Score on the AQLQ quality of life questionnaire (1-7) of >6.   *^i^https://bibliopro.org/media/upload/pdf/descargables/descripcion_aqlq_bibliopro_2009.pdf/AQLQ (Asthma Quality of Life Questionnaire)* | 1 | 2 | | 3 | 4 | 5 | 6 | 7 | 8 | | 9 |

### 4.2 Criteria for partial control of allergic asthma

P4.2.1 **Considering patients undergoing treatment with SAIT, regardless of concomitant basic pharmacological treatment and referring to the allergen(s) for which the patient is undergoing treatment, indicate your degree of agreement that the following items constitute the definition of PARTIAL CONTROL of asthma, as set out in the GEMA 5.4 guideline, applicable to ALLERGIC ASTHMA** ***(where 1 = strongly disagree and 9 = strongly agree)***

| Indicators for defining PARTIAL CONTROL of ALLERGIC ASTHMA | **Strongly disagree** | |  | | | | | | | **Strongly agree** | |
| --- | --- | --- | --- | --- | --- | --- | --- | --- | --- | --- | --- |
| 1. *Any of the following criteria must be met:*  - Daytime symptoms > 2 days per month - Any limitation of activities - Any night-time symptoms - Use of rescue medication > 2 days per month - Lung function:   - 1. FEV1 < 80% of predicted value     2. PEF <80% of personal best - Exacerbations: 1 or more per year | 1 | 2 | | 3 | 4 | 5 | 6 | 7 | 8 | | 9 |

[IF THE PANELLIST INDICATES 1, 2 OR 3, THE FOLLOWING QUESTION WILL APPEAR]: You have indicated that **you** disagree that the partial control criteria based on the GEMA 5.4 guideline **are applicable to allergic asthma**. Please could you give **detailed reasons** for your disagreement with this definition?

_________________________________ (open question)

P4.2.2 **Thinking about patients undergoing treatment with SAIT, regardless of concomitant baseline pharmacological treatment and referring to the allergen(s) for which the patient is undergoing treatment, indicate your level of agreement that the following items (referring to the use of PROMs) also constitute the definition of PARTIAL CONTROL of asthma (where 1 = strongly disagree and 9 = strongly agree)**

| Additional indicators to define PARTIAL CONTROL of ALLERGIC ASTHMA | **Strongly disagree** | |  | | | | | | | **Strongly agree** | |
| --- | --- | --- | --- | --- | --- | --- | --- | --- | --- | --- | --- |
| 1. Allergic asthma symptoms measured with a score of 4 to 7 cm on the VAS scale (0-10).   *^i^ VAS (visual analogue scale)* | 1 | 2 | | 3 | 4 | 5 | 6 | 7 | 8 | | 9 |
| 1. ACT questionnaire score (5-25) from 16 to 19.   *^i^https://www.svnpar.es/wp-content/uploads/2018/11/Test_ACT.pdf / ACT (Asthma Control Test)* | 1 | 2 | | 3 | 4 | 5 | 6 | 7 | 8 | | 9 |
| 1. Score on the AQLQ quality of life questionnaire (1-7) from 4 to 6.   *^i^https://bibliopro.org/media/upload/pdf/descargables/descripcion_aqlq_bibliopro_2009.pdf / AQLQ (Asthma Quality of Life Questionnaire)* | 1 | 2 | | 3 | 4 | 5 | 6 | 7 | 8 | | 9 |

### 4.3 Criteria for poor control of allergic asthma

P4.3.**1 Considering patients undergoing treatment with SAIT, regardless of concomitant basic pharmacological treatment and referring to the allergen(s) for which the patient is undergoing treatment, indicate your degree of agreement that the following item constitutes the definition of POOR CONTROL of asthma, as set out in the GEMA 5.4 guideline, applicable to ALLERGIC ASTHMA *(where 1 = strongly disagree and 9 = strongly agree)***

| Indicators for defining POOR CONTROL of ALLERGIC ASTHMA | **Strongly disagree** | |  | | | | | | | **Strongly agree** | |
| --- | --- | --- | --- | --- | --- | --- | --- | --- | --- | --- | --- |
| - Three or more characteristics of partially controlled asthma are present | 1 | 2 | | 3 | 4 | 5 | 6 | 7 | 8 | | 9 |

[IF THE PANELLIST INDICATES 1, 2 OR 3, THE FOLLOWING QUESTION WILL APPEAR]: You have indicated that **you** disagree that the criteria for poor control based on **the GEMA 5.4 guideline are applicable to allergic asthma**. Please could you give **detailed reasons** for your disagreement with this definition?

_________________________________(open question)

P4.3.2 **Thinking about patients undergoing treatment with SAIT, regardless of concomitant baseline pharmacological treatment and referring to the allergen(s) for which the patient is undergoing treatment, indicate your level of agreement that the following items (referring to the use of PROMs) also constitute the definition of POOR CONTROL of asthma (where 1 = strongly disagree and 9 = strongly agree)**

| Additional indicators for defining POOR CONTROL of ALLERGIC ASTHMA | **Strongly disagree** | |  | | | | | | | **Strongly agree** | |
| --- | --- | --- | --- | --- | --- | --- | --- | --- | --- | --- | --- |
| 1. Allergic asthma symptoms measured with a score of >7 cm on the VAS scale (0-10)   *^i^VAS (visual analogue scale)* | 1 | 2 | | 3 | 4 | 5 | 6 | 7 | 8 | | 9 |
| 1. ACT questionnaire score (5-25) ≤15.   *^i^https://www.svnpar.es/wp-content/uploads/2018/11/Test_ACT.pdf // ACT (Asthma Control Test)* | 1 | 2 | | 3 | 4 | 5 | 6 | 7 | 8 | | 9 |
| 1. Score on the AQLQ quality of life questionnaire (1-7) <4.   *^i^https://bibliopro.org/media/upload/pdf/descargables/descripcion_aqlq_bibliopro_2009.pdf* | 1 | 2 | | 3 | 4 | 5 | 6 | 7 | 8 | | 9 |

### 4.4 Criteria for clinical remission of allergic asthma

In this section, we will ask you about **the criteria for clinical remission of allergic asthma** in patients undergoing treatment with SAIT. Please note that these criteria refer to the allergen(s) for which the patient is undergoing treatment and that, in order to assess remission, the patient must not require concomitant basic pharmacological treatment. Think in general terms and do not take into account the duration of remission, as we will address this aspect in *Section 5: General concepts*.

Q4.4.1 **Thinking about patients undergoing treatment with SAIT and referring to the allergen(s) for which the patient is undergoing treatment, indicate your degree of agreement that the following items constitute the definition of CLINICAL REMISSION of asthma, as set out in the GEMA 5.4 guideline and REMission in ASthma (REMAS) consensus, applicable to ALLERGIC ASTHMA** ***(where 1 = strongly disagree and 9 = strongly agree)***

| Indicators for defining CLINICAL REMISSION of ALLERGIC ASTHMA | **Strongly disagree** | |  | | | | | | | **Strongly agree** | |
| --- | --- | --- | --- | --- | --- | --- | --- | --- | --- | --- | --- |
| - Controlled asthma (ACT ≥ 20). - No need for relief or rescue medication. - No exacerbations and no need for systemic steroid cycles or maintenance treatment. - Spirometry with FEV1 ≥ 80%, or in previous tests, values > 90% of your personal best. - Spirometry with negative bronchodilator test. | 1 | 2 | | 3 | 4 | 5 | 6 | 7 | 8 | | 9 |

[IF THE PANELLIST SELECTS 1, 2 OR 3, THE FOLLOWING QUESTION WILL APPEAR]: You have indicated that you disagree that the clinical referral criteria based on the GEMA 5.4 guideline **are applicable to allergic asthma**. Please could you explain **in detail your reasons** for disagreeing with this definition?

_________________________________ (open question)

## Section 5: General concepts

The aim of this last section is **to address aspects related to the remission of** **allergic diseases** (allergic rhinitis, allergic conjunctivitis and/or allergic asthma ) in patients undergoing treatment with SAIT, referring to the allergen(s) for which they are being treated and thinking in general terms.

### 5.1 Patient satisfaction with SAIT treatment

5.1.1 **Patient satisfaction with the treatment received is directly related to the control and/or remission of the disease.**

1. Please indicate your level of agreement with the usefulness of using the ESPIA questionnaire (satisfaction scale for patients receiving allergen immunotherapy ) to assess patient satisfaction with SAIT in clinical practice:

^i^ https://www.jacionline.org/article/S0091-6749(12)01976-8/fulltext

| **Not useful** |  | | | | | | | **Totally useful** |
| --- | --- | --- | --- | --- | --- | --- | --- | --- |
| 1 | 2 | 3 | 4 | 5 | 6 | 7 | 8 | 9 |

### 5.2 Required duration of clinical remission criteria for allergic diseases (allergic rhinitis, allergic conjunctivitis and/or allergic asthma)

P5.2.1 **Thinking about patients undergoing treatment with SAIT, referring to the allergen(s) for which the patient is undergoing treatment and taking into account that remission may occur for one of the diseases (allergic rhinitis and/or allergic conjunctivitis and/or allergic asthma) and not for others, indicate your level of agreement with the following statements related to the REQUIRED DURATION of the criteria for CLINICAL REMISSION for ALLERGIC DISEASES (allergic rhinitis, allergic conjunctivitis and/or allergic asthma) *(where 1 = strongly disagree and 9 = strongly agree)***

| Required duration of CLINICAL REMISSION criteria while undergoing treatment with SAIT. | **Strongly disagree** | |  | | | | | | | **Strongly agree** | |
| --- | --- | --- | --- | --- | --- | --- | --- | --- | --- | --- | --- |
| 1. The minimum period of time for assessing clinical remission is different for perennial and seasonal allergens. * | 1 | 2 | | 3 | 4 | 5 | 6 | 7 | 8 | | 9 |
| 1. In the case of seasonal allergens, clinical remission criteria must be met for at least one season. *   [IF THE PANELLIST INDICATES 1, 2 OR 3, THE FOLLOWING QUESTION WILL APPEAR]: You have indicated that you disagree with the previous statement. If your disagreement is related to seasonality, what would be the appropriate seasonality for you? | 1 | 2 | | 3 | 4 | 5 | 6 | 7 | 8 | | 9 |
| 1. In the case of perennial allergens, the criteria for clinical remission must be met for at least 6 months. *   [IF THE PANELLIST INDICATES 1, 2 OR 3, THE FOLLOWING QUESTION WILL APPEAR]: You have indicated that you disagree with the previous statement. If your disagreement is related to timing, what would be the appropriate timing for you? | 1 | 2 | | 3 | 4 | 5 | 6 | 7 | 8 | | 9 |

Questions included in wave 2

**As you can see in the graph above, no consensus was reached in Wave 1 on the timing of clinical remission. The frequencies most suggested by the experts participating in Wave 1 were:**

- *At least 2 seasons for seasonal allergens;*
- *At least 1 year for perennial allergens.*

***Please express your level of agreement that the following criteria should be part of the definition of clinical remission.***

| Duration of CLINICAL REMISSION criteria while undergoing treatment with SAIT. | **Strongly disagree** | |  | | | | | | | **Strongly agree** | |
| --- | --- | --- | --- | --- | --- | --- | --- | --- | --- | --- | --- |
| 1. In the case of SEASONAL allergens, the criteria for clinical remission must be met for **at least 2 seasons**. *   [IF THE PANELLIST INDICATES 1, 2 OR 3, THE FOLLOWING QUESTION WILL APPEAR]: You have indicated that you disagree with the previous statement. If your disagreement is related to Required duration , what would be the appropriate Required duration for you? | 1 | 2 | | 3 | 4 | 5 | 6 | 7 | 8 | | 9 |
| 1. In the case of PERENNIAL allergens, the criteria for clinical remission must be met for **at least 1 year**. *   [IF THE PANELLIST INDICATES 1, 2 OR 3, THE FOLLOWING QUESTION WILL APPEAR]: You have indicated that you disagree with the previous statement. If your disagreement is related to timing, what would be the appropriate timing for you? |  |  | |  |  |  |  |  |  | |  |

### 5.3 Overall remission of allergic diseases (allergic rhinitis, allergic conjunctivitis and/or allergic asthma

P5.3.1 **Thinking about patients undergoing treatment with SAIT and referring to the allergen(s) for which the patient is undergoing treatment, indicate your level of agreement with the following definitions related to OVERALL REMISSION for allergic diseases (allergic rhinitis, allergic conjunctivitis and/or allergic asthma) *(where 1 = strongly disagree and 9 = strongly agree)***

| DEFINITION OF OVERALL REMISSION. | **Strongly disagree** | |  | | | | | | | **Strongly agree** | |
| --- | --- | --- | --- | --- | --- | --- | --- | --- | --- | --- | --- |
| 1. Global remission is defined as the clinical remission of all concurrent allergic conditions treated with SAIT. * | 1 | 2 | | 3 | 4 | 5 | 6 | 7 | 8 | | 9 |
| 1. The minimum period of time for assessing overall remission is different for perennial and seasonal allergens. | 1 | 2 | | 3 | 4 | 5 | 6 | 7 | 8 | | 9 |
| 1. In the case of seasonal allergens, the GLOBAL remission criteria must be met for at least 1 season. *   [IF THE PANELLIST INDICATES 1, 2 OR 3, THE FOLLOWING QUESTION WILL APPEAR]: You have indicated that you disagree with the previous statement. If your disagreement is related to seasonality, what would be the appropriate seasonality for you? | 1 | 2 | | 3 | 4 | 5 | 6 | 7 | 8 | | 9 |
| 1. In the case of perennial allergens, the GLOBAL remission criteria must be met for at least 6 months.*   [IF THE PANELLIST INDICATES 1, 2 OR 3, THE FOLLOWING QUESTION WILL APPEAR]: You have indicated that you disagree with the previous statement. If your disagreement is related to timing, what would be the appropriate timing for you? | 1 | 2 | | 3 | 4 | 5 | 6 | 7 | 8 | | 9 |

Questions included in wave 2

**As you can see in the graph above, no consensus was reached in Wave 1 on the timing of global remission. The frequencies most suggested by the experts participating in Wave 1 were:**

- *At least 2 seasons for seasonal allergens;*
- *At least one year for perennial allergens.*

***Please indicate your level of agreement that the following criteria should be part of the definition of global remission.***

| DEFINITION OF OVERALL REMISSION. | **Strongly disagree** | |  | | | | | | | **Strongly agree** | |
| --- | --- | --- | --- | --- | --- | --- | --- | --- | --- | --- | --- |
| 1. In the case of SEASONAL allergens, the GLOBAL remission criteria must be met for **at least 2 seasons.**   [IF THE PANELLIST INDICATES 1, 2 OR 3, THE FOLLOWING QUESTION WILL APPEAR]: You have indicated that you disagree with the above statement. If your disagreement is related to seasonality, what would be the appropriate seasonality for you? | 1 | 2 | | 3 | 4 | 5 | 6 | 7 | 8 | | 9 |
| 1. In the case of PERENNIAL allergens, the GLOBAL remission criteria must be met for **at least 1 year**.   [IF THE PANELLIST INDICATES 1, 2 OR 3, THE FOLLOWING QUESTION WILL APPEAR]: You have indicated that you disagree with the above statement. If your disagreement is related to timing, what would be the appropriate timing for you? | 1 | 2 | | 3 | 4 | 5 | 6 | 7 | 8 | | 9 |

### 5.4 Long-term remission of allergic diseases (allergic rhinitis, allergic conjunctivitis and/or allergic asthma)

P5.4.1 **Thinking about patients who have completed treatment with SAIT and referring to the allergen(s) for which the patient is being treated, indicate your level of agreement that the following items constitute the definition of LONG-TERM remission for allergic diseases (allergic rhinitis, allergic conjunctivitis and/or allergic asthma) *(where 1 = strongly disagree and 9 = strongly agree)***

| DEFINITION OF LONG-TERM REMISSION. | **Strongly disagree** | |  | | | | | | | **Strongly agree** | |
| --- | --- | --- | --- | --- | --- | --- | --- | --- | --- | --- | --- |
| 1. Long-term remission is defined as the maintenance of clinical remission of one or more concurrent allergic diseases 1 year after the end of treatment with SAIT.*   [IF THE PANELLIST INDICATES 1, 2 OR 3, THE FOLLOWING QUESTION WILL APPEAR]: You have indicated that you disagree with the above statement. If your disagreement is related to timing, what would be the appropriate timing for you? | 1 | 2 | | 3 | 4 | 5 | 6 | 7 | 8 | | 9 |
| 1. Long-term remission is defined as the maintenance of clinical remission of one or more concurrent allergic diseases 3 years after the end of treatment with SAIT.*   [IF THE PANELLIST INDICATES 1, 2 OR 3, THE FOLLOWING QUESTION WILL APPEAR]: You have indicated that you disagree with the above statement. If your disagreement is related to timing, what would be the appropriate timing for you? | 1 | 2 | | 3 | 4 | 5 | 6 | 7 | 8 | | 9 |
