# Supplementary material for: Criteria for Control and Remission of Respiratory Allergic Disease With Allergen Immunotherapy: A Delphi Consensus
Source: Clin Transl Allergy. 2026 Aug 2;16(8):e70191. doi: 10.1002/clt2.70191 (PMC13429802; doi:10.1002/clt2.70191)
Supplement: Supplementary file 2 — Supporting Information S2 [file CLT2-16-e70191-s003.pptx]

## Slide 1
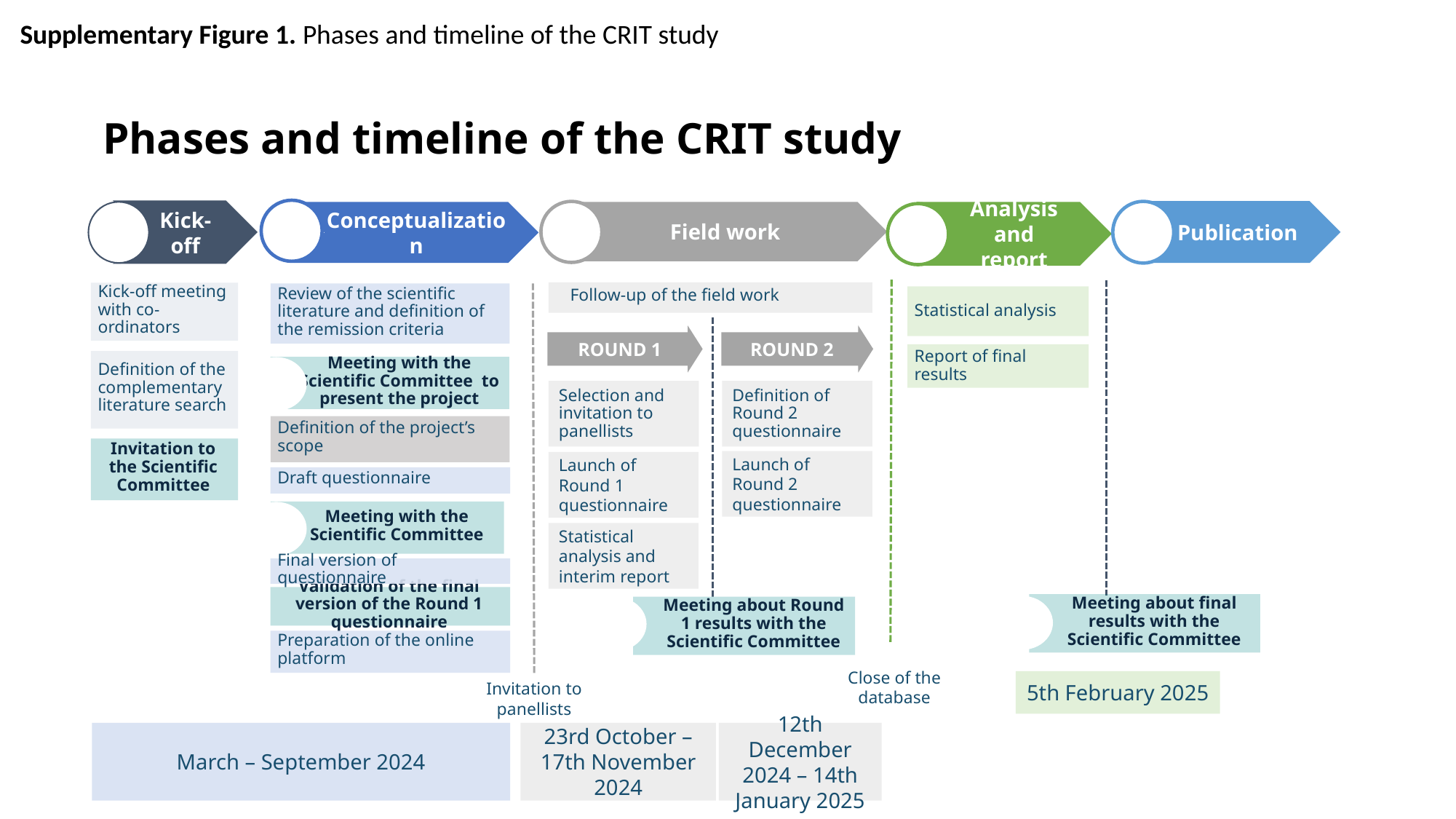

Supplementary Figure 1. Phases and timeline of the CRIT study
Phases and timeline of the CRIT study
Publication
Kick-off
Analysis and report
Conceptualization
Field work
Follow-up of the field work
Kick-off meeting with co-ordinators
Review of the scientific literature and definition of the remission criteria
Statistical analysis
ROUND 1
ROUND 2
Report of final results
Definition of the complementary literature search
Meeting with the Scientific Committee to present the project
Selection and invitation to panellists
Definition of Round 2 questionnaire
Definition of the project’s scope
Invitation to the Scientific Committee
Launch of Round 2 questionnaire
Launch of Round 1 questionnaire
Draft questionnaire
Meeting with the Scientific Committee
Statistical analysis and interim report
Final version of questionnaire
Validation of the final version of the Round 1 questionnaire
Meeting about final results with the Scientific Committee
Meeting about Round 1 results with the Scientific Committee
Preparation of the online platform
Close of the database
5th February 2025
Invitation to panellists
March – September 2024
23rd October – 17th November 2024
12th December 2024 – 14th January 2025

## Slide 2
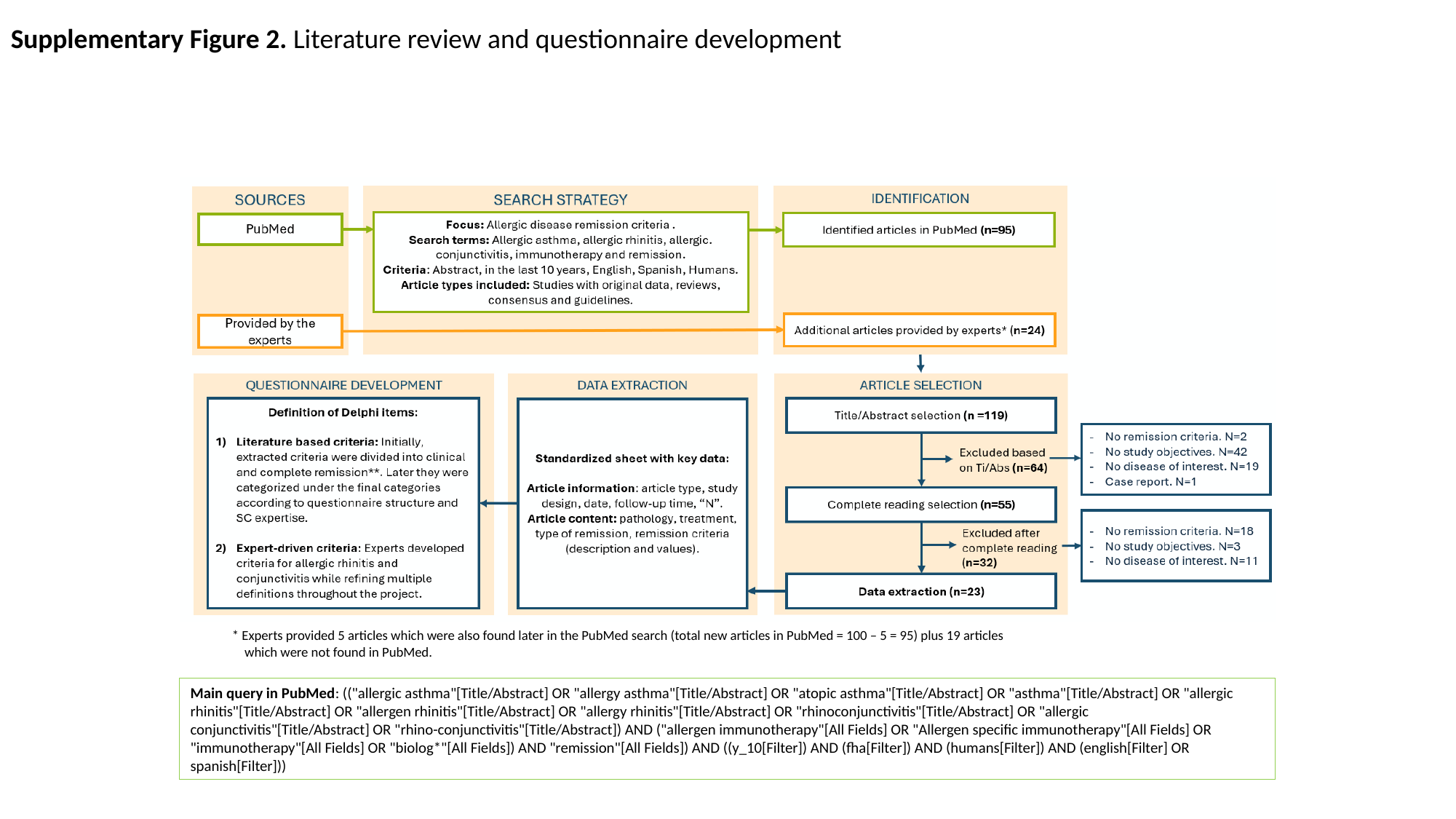

Supplementary Figure 2. Literature review and questionnaire development
* Experts provided 5 articles which were also found later in the PubMed search (total new articles in PubMed = 100 – 5 = 95) plus 19 articles
 which were not found in PubMed.
Main query in PubMed: (("allergic asthma"[Title/Abstract] OR "allergy asthma"[Title/Abstract] OR "atopic asthma"[Title/Abstract] OR "asthma"[Title/Abstract] OR "allergic rhinitis"[Title/Abstract] OR "allergen rhinitis"[Title/Abstract] OR "allergy rhinitis"[Title/Abstract] OR "rhinoconjunctivitis"[Title/Abstract] OR "allergic conjunctivitis"[Title/Abstract] OR "rhino-conjunctivitis"[Title/Abstract]) AND ("allergen immunotherapy"[All Fields] OR "Allergen specific immunotherapy"[All Fields] OR "immunotherapy"[All Fields] OR "biolog*"[All Fields]) AND "remission"[All Fields]) AND ((y_10[Filter]) AND (fha[Filter]) AND (humans[Filter]) AND (english[Filter] OR spanish[Filter]))

## Slide 3
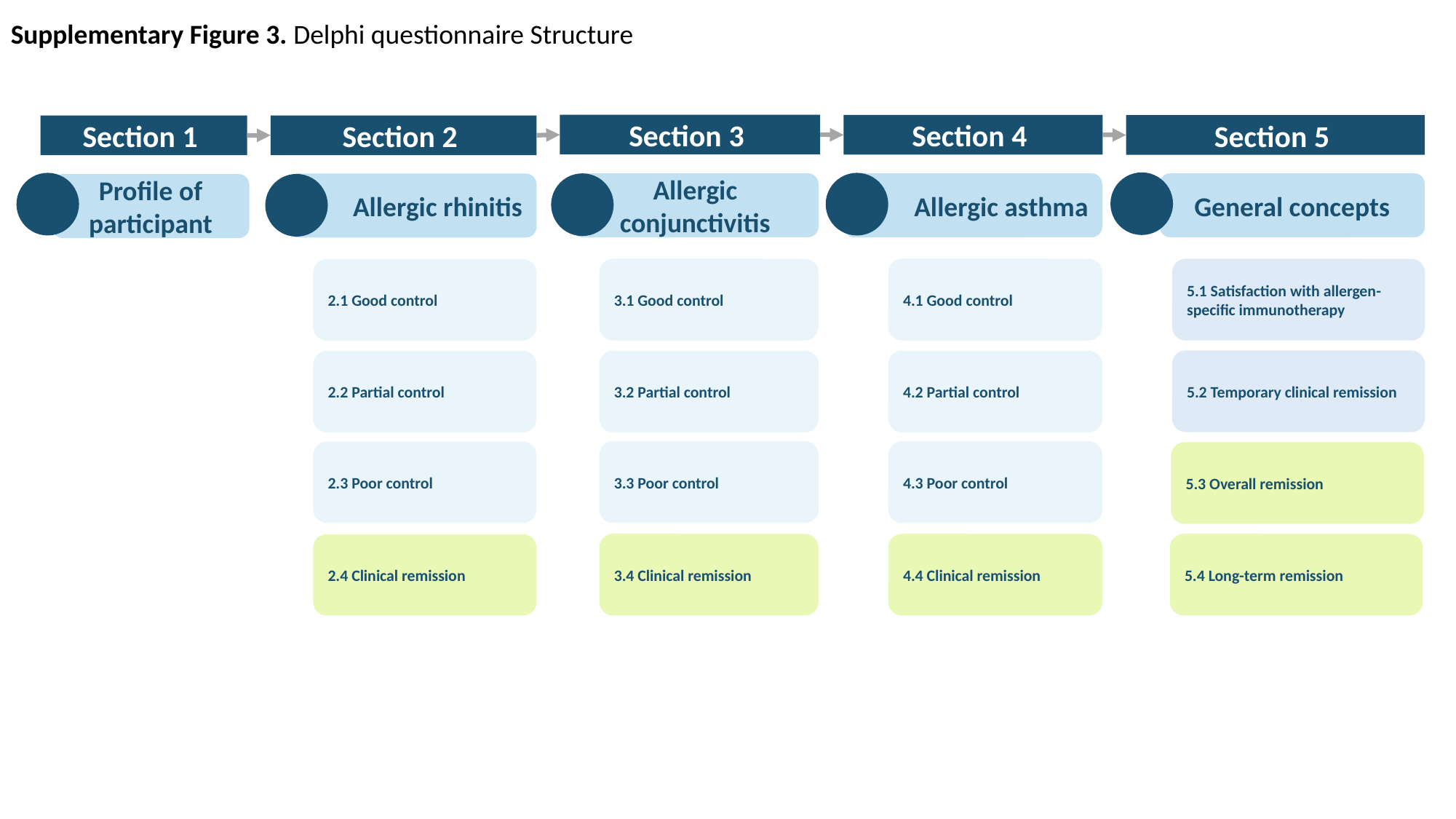

Supplementary Figure 3. Delphi questionnaire Structure
Section 3
Section 4
Section 5
Section 2
Section 1
Allergic conjunctivitis
Allergic asthma
General concepts
Allergic rhinitis
Profile of participant
3.1 Good control
4.1 Good control
5.1 Satisfaction with allergen-specific immunotherapy
2.1 Good control
5.2 Temporary clinical remission
3.2 Partial control
4.2 Partial control
2.2 Partial control
3.3 Poor control
4.3 Poor control
2.3 Poor control
5.3 Overall remission
3.4 Clinical remission
4.4 Clinical remission
5.4 Long-term remission
2.4 Clinical remission

## Slide 4
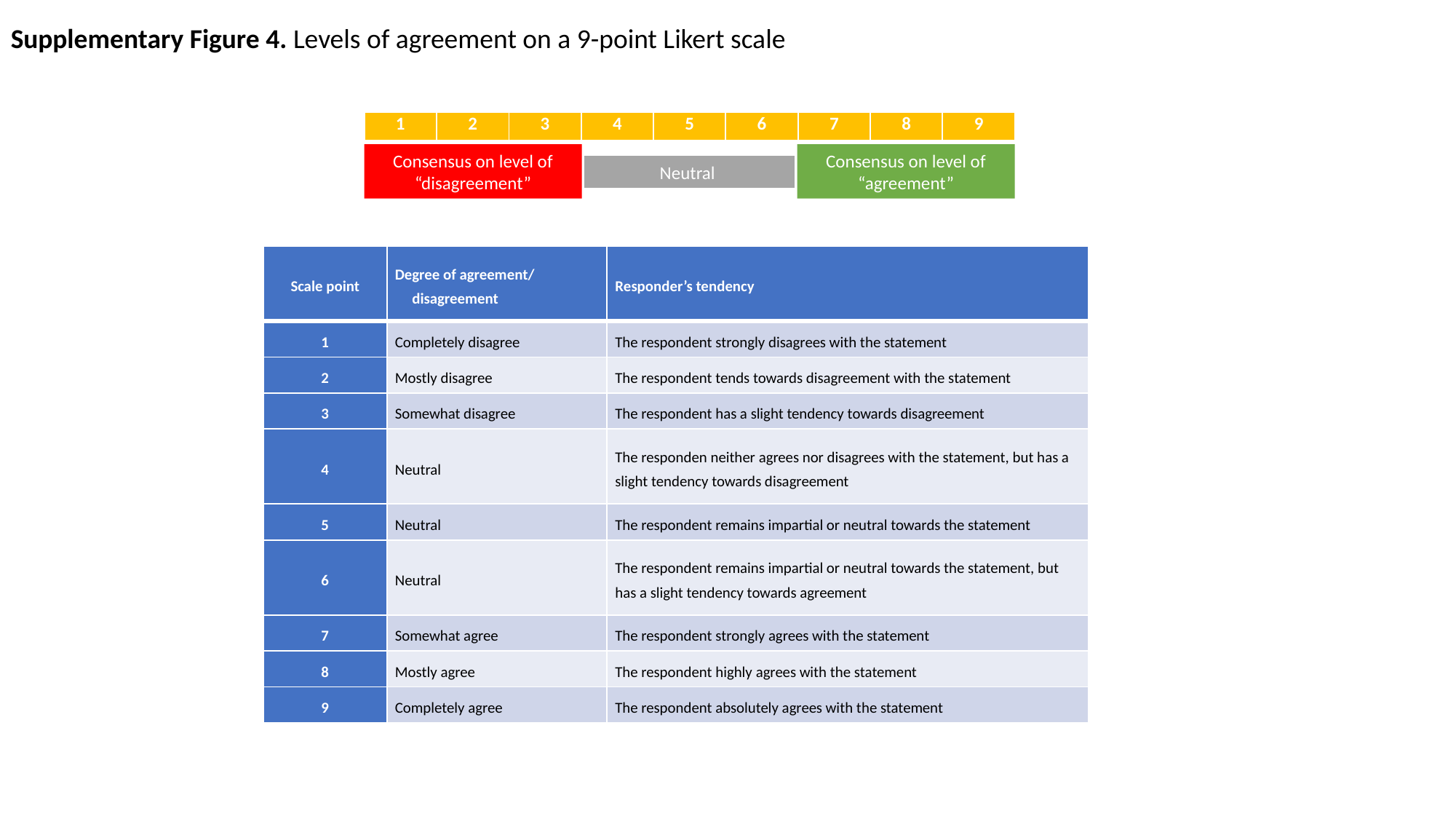

Supplementary Figure 4. Levels of agreement on a 9-point Likert scale
| 1 | 2 | 3 | 4 | 5 | 6 | 7 | 8 | 9 |
| --- | --- | --- | --- | --- | --- | --- | --- | --- |
Consensus on level of “disagreement”
Consensus on level of “agreement”
Neutral
| Scale point | Degree of agreement/ disagreement | Responder’s tendency |
| --- | --- | --- |
| 1 | Completely disagree | The respondent strongly disagrees with the statement |
| 2 | Mostly disagree | The respondent tends towards disagreement with the statement |
| 3 | Somewhat disagree | The respondent has a slight tendency towards disagreement |
| 4 | Neutral | The responden neither agrees nor disagrees with the statement, but has a slight tendency towards disagreement |
| 5 | Neutral | The respondent remains impartial or neutral towards the statement |
| 6 | Neutral | The respondent remains impartial or neutral towards the statement, but has a slight tendency towards agreement |
| 7 | Somewhat agree | The respondent strongly agrees with the statement |
| 8 | Mostly agree | The respondent highly agrees with the statement |
| 9 | Completely agree | The respondent absolutely agrees with the statement |
