# Supplementary material for: Criteria for Control and Remission of Respiratory Allergic Disease With Allergen Immunotherapy: A Delphi Consensus
Source: Clin Transl Allergy. 2026 Aug 2;16(8):e70191. doi: 10.1002/clt2.70191 (PMC13429802; doi:10.1002/clt2.70191)
Supplement: Supplementary file 3 — Table S1: Methodological plan guiding the Delphi consensus process. [file CLT2-16-e70191-s002.docx]

**Supplementary Table 1.** Methodological plan guiding the Delphi consensus process

|  | **Description applied in the current study** |
| --- | --- |
| **Background/Rationale** | Despite the availability of multiple validated tools to assess symptoms, quality of life, and disease activity in respiratory allergic diseases, there is a lack of standardized, consensus-based criteria to define clinical control and remission in patients undergoing allergen immunotherapy. This heterogeneity limits comparability across studies and creates uncertainty in routine clinical practice. A Delphi consensus was therefore undertaken to synthesize expert opinion and propose structured criteria to support assessment in real-world settings |
| **Study objective** | To reach expert consensus on criteria for control and remission in respiratory allergic diseases (allergic rhinitis, conjunctivitis, and asthma) in patients undergoing allergen immunotherapy |
| **Study design** | Modified Delphi consensus study with two structured online rounds |
| **Reporting guidance** | Conducted and reported in accordance with DELPHISTAR recommendations |
| **Expert panel eligibility criteria** | Pre-defined criteria including clinical experience in respiratory allergy, involvement in allergen immunotherapy, and scientific or clinical expertise |
| **Panel recruitment** | Invitation through the SEAIC Immunotherapy Committee; voluntary participation |
| **Number of rounds** | Two pre-planned Delphi rounds |
| **Questionnaire development** | Based on a structured narrative literature review and iterative refinement by the Scientific Committee |
| **Consensus definition** | Consensus defined a priori as ≥70% agreement within pre-defined Likert scale ranges |
| **Feedback between rounds** | Aggregated percentages of agreement, disagreement, and neutral responses provided to panellists |
| **Anonymity** | Full anonymity of participants was ensured throughout the Delphi process |
| **Data analysis** | Descriptive analysis of agreement levels; comparison between rounds for re-assessed items |
| **Ethical considerations** | Expert survey without patient involvement; informed consent obtained electronically |
| **Sponsor involvement** | Sponsor provided funding and logistical support only; it had no involvement in scientific decisions |
