# Supplementary material for: Criteria for Control and Remission of Respiratory Allergic Disease With Allergen Immunotherapy: A Delphi Consensus
Source: Clin Transl Allergy. 2026 Aug 2;16(8):e70191. doi: 10.1002/clt2.70191 (PMC13429802; doi:10.1002/clt2.70191)
Supplement: Supplementary file 4 — Table S2: DELPHISTAR reporting checklist for Delphi studies. [file CLT2-16-e70191-s001.docx]

**Supplementary Table 2**. DELPHISTAR reporting checklist for Delphi studies

| **Topic** | **Section** | **Item** | **Checklist Item** | **Location where item is reported** | **Wording** |
| --- | --- | --- | --- | --- | --- |
| **I**  **Title and Abstract** |  | 1 | Identification as a Delphi study in the title | Page 1 | Criteria for control and remission of respiratory allergic disease with allergen immunotherapy: A Delphi Consensus |
|  |  | 2 | Identification as a Delphi study in the abstract | Page 7 | Forty allergists with ≥5 years of clinical experience participated in a two-round Delphi study. |
|  |  | 3 | Structured abstract | Page 7 | Background, methods, results and conclusions |
| **II**  **Context** | **Formal** | 4 | Information about the sources of funding | Pages 3, 9 | Funding: This project was supported by an unrestricted research grant from Allergy Therapeutics Ibérica, S.L.U. to Spanish Society of Allergy and Clinical Immunology (SEAIC). |
|  |  | 5 | Information about the team of authors and/or researchers (e.g., discipline, institution) | Page 11 | The SC comprised 12 allergologists, most of whom had more than 20 years of experience in immunotherapy from three SEAIC committees: Immunotherapy, Rhinology and Allergic Conjunctivitis, and Asthma. |
|  |  | 6 | Information about method consulting | Page 11 | The questionnaire was developed by the SC following a review of the scientific literature on disease control and remission in allergic respiratory conditions, including rhinitis, conjunctivitis, and asthma, |
|  |  | 7 | Information about the project background | Page 9 | This project was conceived and organized by members of the Immunotherapy Committee of the Spanish Society of Allergy and Clinical Immunology (SEAIC) and conducted as part of its official scientific activities. Conceptualization and study design, including the development of the questionnaire, were driven exclusively by the members of the Scientific Committee (SC) (most of whom are allergists with more than 20 years of experience in immunotherapy), following approval by the SEAIC Executive Board as an official scientific activity. |
|  |  | 8 | Information about the study protocol | Page 10 | A modified classic Delphi study was carried out, following the methodological plan described in Supplementary Table 1. |
|  | **Content** | 9 | Justification of the chosen method (Delphi) to answer the research question | Page 10 | The Delphi method was selected as the most appropriate approach by which to address the study’s objective, as it allows the systematic collection and structured synthesis of expert judgments in areas where empirical evidence is limited or heterogeneous or conflicting. In the context of respiratory allergic diseases, particularly regarding criteria for disease control and remission in patients undergoing AIT, the available literature is scarce and lacks standardized definitions. The Delphi is a widely accepted and structured approach technique that therefore provides a rigorous and transparent preliminary framework by which to identify areas of agreement and disagreement among experts and propose consensus-based criteria to support clinical assessment. |
|  |  | 10 | Aim of the Delphi study (e.g., consensus, forecasting) | Page 9 | So, the objective of this study was to explore a possible unified definition of control and remission for allergic rhinitis, conjunctivitis, and asthma in patients receiving AIT in routine clinical practice using Delphi process to explore the consensus reached on these criteria by a group of experts. These proposed criteria are intended as an initial exploratory framework to support clinical evaluation and discussion. Further refinement and validation are required before they can be considered for routine clinical use. |
| **III**  **Method** | **Body & Integration of knowledge** | 11 | Identification and elucidation of relevant expertise, spheres of experience, and perspectives (e.g., theory, practice, affected groups, disciplines) | Page 11 | The panellist represented clinical practice–based expertise in respiratory allergic diseases and AIT, contributing perspectives derived from routine patient management, long-term follow-up, and the practical use of symptom-based and disease-control assessment tools in real-world clinical settings. |
|  |  | 12 | Handling of knowledge, expertise and perspectives which are missing or have been deliberately not integrated | Page 16 | The Delphi panel was intentionally composed of experienced allergists, as AIT is a specialized treatment administered by physicians with specific expertise in allergic diseases. Consequently, the consensus reflects the perspective of clinicians with established expertise in allergies and AIT. Patient representatives were not included, as the aim was to establish clinical guidance definitions based on expert knowledge and current clinical practice. Another limitation is that the study was conducted in a single country, which may limit generalizability, and therefore external validation in different health systems is necessary, as already mentioned. |
|  |  | 13 | Basic definition of expert^1^ | Page 11 | The expert panel consisted of 40 specialists with a minimum of 5 years of post-specialization clinical experience in allergy. |
|  | **Delphi variant and modifications** | 14 | Identification of the type of Delphi variant and potential modifications (e.g., classic Delphi, real-time Delphi, group Delphi) | Page 10 | A modified classic Delphi study was carried out, following the methodological plan described in Supplementary Table 1…  …The study was performed in three phases: 1) From March to September 2024, an online meeting of the SC, composed of 12 members, to discuss the problem, establish the project’s scope and draft the Delphi questionnaire. 2) Two successive rounds of anonymous online structured questionnaires with aggregated feedback from panel of experts between rounds to obtain their opinion [35,36]: 2A) The first round (from 23 October, 2024 to 17 November, 2024); 2B) The intermediate analysis; 2C) The second round (from 12 December, 2024 to 14 January, 2025). 3) Analysis and discussion of the results with the SC to reach conclusions (February 2025) (Supplementary Figure 1). |
|  |  | 15 | Justification of the Delphi variant and modifications, including during the Delphi study, if applicable | Page 12 | This Delphi consensus was conducted in two structured and predefined rounds of questions.  After completion of the first Delphi round, participants were provided with aggregated feedback summarising the group responses for each item, including the percentages of agreement, disagreement, and neutral responses. Feedback was aggregated across all participants and not differentiated by expert subgroups. The second Delphi round was designed to further explore areas of disagreement by re-assessing items that did not reach consensus in the first round. The questions on which no consensus (5 items) was reached (<70% agreement) were repeated in the second round to explore certain disagreements further (Supplementary Material-Questionnaires). Two more items were included in the second round of questions to prioritise definitions with the highest impact on clinical practice |
|  | **Sample of experts** | 16 | Selection criteria for the experts (per round, per expert group if applicable) | Page 11 | An initial invitation to participate in the Delphi survey was disseminated through the SEAIC Immunotherapy Committee. Potential experts were informed about the objectives and procedures of the study, drawing on the Committee’s membership of allergists actively involved in the clinical management of respiratory allergic diseases and the prescription of AIT. Participation was voluntary. Eligibility was subsequently confirmed through predefined screening criteria embedded in the online questionnaire to ensure appropriate clinical expertise. All experts fulfilling the eligibility criteria who completed the first round were invited to participate in the subsequent round. The screening criteria and eligibility questions are detailed in the questionnaire (Supplementary Material-Questionnaires). |
|  |  | 17 | Identification of the experts | Page 11 | Potential experts were informed about the objectives and procedures of the study, drawing on the Committee’s membership of allergists actively involved in the clinical management of respiratory allergic diseases and the prescription of AIT. |
|  |  | 18 | Information about recruiting and any subsequent recruiting of experts | Page 11 | Participation was voluntary. Eligibility was subsequently confirmed through predefined screening criteria embedded in the online questionnaire to ensure appropriate clinical expertise. All experts who met the eligibility criteria and completed the first round were invited to participate in the subsequent round. |
|  | **Survey** | 19 | Elucidation of the content development for the questionnaire^2^ | Page 11 | The questionnaire was developed by the SC following a review of the scientific literature on disease control and remission in allergic respiratory conditions, including rhinitis, conjunctivitis, and asthma, with the methodological support of the medical communications consulting firm, Adelphi Targis, SL. An additional strategy search was focused on allergic disease remission criteria, and the search terms were allergic rhinitis, allergic conjunctivitis, allergic asthma, immunotherapy remission. The questionnaire was based on literature criteria (including rating scales) and expert-driven criteria (Supplementary Figure 2). |
|  |  | 20 | Description of the questionnaire (content and structure) | Page 11-12 | The questionnaire was divided into 5 blocks: Section 1 – Participant profile, Section 2 – Allergic rhinitis, Section 3 – Allergic conjunctivitis, Section 4 – Allergic asthma, Section 5 – General concepts (Supplementary Figure 3). Criteria included in the Delphi questionnaire incorporate daytime/nocturnal symptoms, activity limitation, symptomatic medication use, exacerbations, and both subjective/objective measures: unified VAS (0–10), control questionnaires (RCAT, ACT), quality-of-life scores (RQLQ, AQLQ), nasal PNIF, and Efron scale for conjunctival hyperaemia. For asthma, GEMA/GINA control criteria were retained, supplemented with ACT, AQLQ, and FEV1 for consistency. |
|  | **Delphi rounds** | 21 | Number of Delphi rounds | Page 12 | This Delphi consensus was conducted in two structured and predefined rounds of questions. |
|  |  | 22 | Information about the aims of the individual Delphi rounds | Page 12 | The first Delphi round aimed to collect expert opinions on pre-defined criteria for control and remission in respiratory allergic diseases, and to assess the initial level of agreement on all proposed items. It comprised 15 descriptive items in Section 1 and 72 items on a Likert-type scale in Sections 2 to 5, with some associated open-ended questions. After completion of the first Delphi round, participants were provided with aggregated feedback summarising the group responses for each item, including the percentages of agreement, disagreement, and neutral responses. Feedback was aggregated across all participants and not differentiated by expert subgroups. The second Delphi round was designed to further explore areas of disagreement by re-assessing items that did not reach consensus in the first round. |
|  |  | 23 | Disclosure and justification of the criterion for discontinuation | Page 12 | The number of Delphi rounds was predefined prior to study initiation. In accordance with established Delphi methodology, which commonly involves two to three iterative rounds with analysis following each wave, two rounds were planned. After completion of the second round, a high level of consensus had been achieved across the proposed criteria, and no substantial new areas of disagreement emerged. Therefore, the predefined number of rounds was considered sufficient to address the study objectives, and no additional rounds were deemed necessary. |
|  | **Feedback** | 24 | Information about what data was reported back per round | Page 12 | After completion of the first Delphi round, participants were provided with aggregated feedback summarising the group responses for each item, including the percentages of agreement, disagreement, and neutral responses. |
|  |  | 25 | Information on how the results of the previous Delphi round were fed back to the experts surveyed (e.g., via frequencies, mean values, measures of dispersion, listing of comments) | Page 12 | After completion of the first Delphi round, participants were provided with aggregated feedback summarising the group responses for each item, including the percentages of agreement, disagreement, and neutral responses. |
|  |  | 26 | Information on whether feedback was differentiated by specific groups (e.g., by field of expertise, institutional affiliation) | Page 12 | Feedback was aggregated across all participants and not differentiated by expert subgroups. |
|  |  | 27 | Information about how dissent and unclear results were handled | Page 12 | The questions on which no consensus (5 items) was reached (<70% agreement) were repeated in the second round |
|  | **Data analysis** | 28 | Disclosure of the quantitative and qualitative analytical strategy | Page 12-13 | Nominal variables were described by means of frequency. For continuous variables, central tendency and dispersion measurement were calculated. |
|  |  | 29 | Definition and measurement of consensus | Page 11 | Consensus on a statement was defined prior to the initiation of both Delphi rounds, as when ≥70% of the respondents scored within the three-point range (1-3 or 7-9). |
|  |  | 30 | Information on group-specific analysis or weighting of experts (e.g., theory vs. practice, discipline-specific analysis) | Page 13 | No group-specific analyses or weighting of expert responses were performed. All participants contributed equally to the analysis, as the panel represented a single professional group with comparable clinical expertise. |
| **IV**  **Results** | **Delphi process** | 31 | Illustration of the Delphi study (e.g., in a flow chart) | Supplementary figure 1 | A summary of the Delphi study is illustrated in a flow chart (Figure 1). |
|  |  | 32 | Information about special aspects during the Delphi study (e.g., deviations from the intended approach with justification) | N/A | No applicable. |
|  |  | 33 | Number of experts per round (both invited and participating) | Page 13 | Initially, 43 experts were invited, 40 of whom completed Round 1 and Round 2 answering all the questions of the survey. |
|  | **Results** | 34 | Presentation of the results for each Delphi round and the final results | Page 13 | In the first round, agreement (score 7-9 on the 9-point Likert scale) was achieved on 67 items (93%) out of the 72 items in Sections 2 to 5. The remaining five items, without agreement, were reevaluated in the second round together with two new items to investigate a further two questions in greater depth. Consensus was ultimately reached in all items after the two rounds (Table 2). Therefore, a final consensus on all 72 items was reached after the two rounds. |
| **V Discussion** | **Quality of findings** | 35 | Highlighting the findings from the Delphi study | Page 15-16 | The central findings can be summarized as follows: Definitions of good, partial, and poor disease control have been proposed for allergic rhinitis, conjunctivitis, and asthma, together with criteria for global and long-term remission. It should be noted that this is the first consensus to explicitly propose remission criteria for allergic rhinitis and conjunctivitis, while maintaining the established criteria for asthma. The proposed control criteria suggest incorporating not only symptoms and medication use, but also symptom scale results, quality-of-life scores, and functional measures. In addition, the consensus supports the value of the ESPIA questionnaire for assessing patients’ satisfaction with AIT…. |
|  |  | 36 | Validity of the results (e.g., transferability of the findings) | Page 16 | Their routine application may pose practical difficulties. The systematic use of questionnaires, functional measurements, and quality-of-life instruments require additional time, organizational resources, and, in some settings, financial investment. These requirements may limit feasibility in high-volume outpatient clinics and should be considered when translating the consensus into daily practice. Nevertheless, it is preferable to have defined criteria that can be used, whenever possible, rather than to lack them. It is important to note that it remains unclear whether the systematic use of these definitions would directly lead to the optimization of treatment strategies or improved patient outcomes. These benefits are currently hypothetical and require confirmation through prospective real-world studies of different healthcare systems, evaluating clinical impact, feasibility, and cost-effectiveness. |
|  |  | 37 | Reliability of the results (e.g., split half, inter-rater reliability) | Page 15 | To support the reliability of the findings, responses were collected and analysed anonymously, and free-text comments were independently reviewed by two researchers. |
|  |  | 38 | Reflection on potential limitations (e.g., number of experts, response bias) | Page 16 | As with all Delphi surveys, the findings reflect expert consensus, which defines their limitations and calls for subsequent confirmation. The Delphi panel was intentionally composed of experienced allergists, as AIT is a specialized treatment administered by physicians with specific expertise in allergic diseases. Consequently, the consensus reflects the perspective of clinicians with established expertise in allergies and AIT. Patient representatives were not included, as the aim was to establish clinical guidance definitions based on expert knowledge and current clinical practice. Another limitation is that the study was conducted in a single country, which may limit generalizability, and therefore external validation in different health systems is necessary, as already mentioned. Nonetheless, the high level of agreement achieved suggests consistency in expert opinion. |

^1^ “Experts” are the participants; these can be people from academia, practice, or representatives of lived experience (e.g., patients, family members).

^2^ The term “questionnaire” stands for the survey instrument regardless of whether quantitative or qualitative items are integrated or weighted.

Adapted from: Niederberger, M., Schifano, J., Deckert, S., Hirt, J., Homberg, A., Köberich, S., et al. Delphi studies in social and health sciences—Recommendations for an interdisciplinary standardized reporting (DELPHISTAR). PLoS One. 2024;19(8):e0304651.
